# A pan-genome of 69 *Arabidopsis thaliana* accessions reveals a conserved genome structure throughout the global species range

**DOI:** 10.1038/s41588-024-01715-9

**Published:** 2024-04-11

**Authors:** Qichao Lian, Bruno Huettel, Birgit Walkemeier, Baptiste Mayjonade, Céline Lopez-Roques, Lisa Gil, Fabrice Roux, Korbinian Schneeberger, Raphael Mercier

**Affiliations:** 1 Department of Chromosome Biology, Max Planck Institute for Plant Breeding Research, Cologne, Germany; 2 Max Planck-Genome-centre Cologne, Max Planck Institute for Plant Breeding Research, Cologne, Germany; 3 Laboratoire des Interactions Plantes-Microbes-Environnement, Institut National de Recherche pour l’Agriculture, l’Alimentation et l’Environnement, CNRS, Université de Toulouse, Castanet-Tolosan, France; 4 INRAE, GeT-PlaGe, Genotoul, Castanet-Tolosan, France; 5 Faculty of Biology, Ludwig-Maximilians-University Munich, Planegg-Martinsried, Germany; 6 Cluster of Excellence on Plant Sciences, Heinrich-Heine University, Düsseldorf, Germany

**Keywords:** Functional genomics, Plant molecular biology, Genomics

## Abstract

Although originally primarily a system for functional biology, *Arabidopsis thaliana* has, owing to its broad geographical distribution and adaptation to diverse environments, developed into a powerful model in population genomics. Here we present chromosome-level genome assemblies of 69 accessions from a global species range. We found that genomic colinearity is very conserved, even among geographically and genetically distant accessions. Along chromosome arms, megabase-scale rearrangements are rare and typically present only in a single accession. This indicates that the karyotype is quasi-fixed and that rearrangements in chromosome arms are counter-selected. Centromeric regions display higher structural dynamics, and divergences in core centromeres account for most of the genome size variations. Pan-genome analyses uncovered 32,986 distinct gene families, 60% being present in all accessions and 40% appearing to be dispensable, including 18% private to a single accession, indicating unexplored genic diversity. These 69 new *Arabidopsis thaliana* genome assemblies will empower future genetic research.

## Abstract (web summary)

A pan-genome of *Arabidopsis thaliana* constructed using chromosome-level genome assemblies of 69 diverse accessions reveals a conserved genome structure throughout the global species range.

## Main

Genome rearrangements can dramatically impact genetic diversity, phenotypes^1,2^, recombination^3–8^ and thus local adaptation and evolution^9–12^. The whole-genome alignment of complete genome assemblies, which can be achieved using long-read Oxford Nanopore Technologies (ONT) and PacBio high-fidelity (HiFi) technologies, facilitates the identification of large and complex structural variants (SVs)^13,14^. Pan-genomes, which aggregate multiple genomes covering the diversity of a given species, provide greater insights into the overall genetic diversity compared to using a single reference and have allowed researchers to determine the natural phenotypic variation of a species^15–18^, as recently shown in plant and animal species, including soybean^19^, tomato^20,21^, potato^22^, rice^23–25^, maize^26^, barley^27^, wheat^28^, apple^29^, silkworm^12^ and human^30,31^.

The first genome sequence of *Arabidopsis thaliana* (Col-0) was released in 2000 (ref. ^32^) and has greatly boosted plant biology and breeding research. This assembly was based on the sequencing of bacterial artificial chromosomes using Sanger technology^32^ and (with some updates) has served as a reference genome until today. Based on this reference genome, several studies applying whole-genome resequencing arrays or short-read sequencing on thousands of worldwide natural accessions of *A. thaliana* (in particular, Africa, Eurasia and North America) have unraveled natural genomic variations, including single-nucleotide polymorphisms (SNPs), small indels and SVs. This, in turn, revealed the evolutionary history, divergence and adaptation of *A. thaliana* at the macro- and micro-evolutionary scales^33–40^. However, only a limited number of large and complex SVs were reported in *Arabidopsis*, including the well-characterized ~1.2 Mb inversion on chromosome 4 between Col-0 and L*er*^5,8,41^ and a ~2.5 Mb inversion on chromosome 3 between Col-0 and Sha^3^, which was captured through chromosome-level assemblies of a few *A. thaliana* accessions^3,5,42–45^. A first *Arabidopsis* pan-genome analysis used the whole-genome assembly of seven accessions based on PacBio CLR, providing a first glimpse into the diversity of this species^3^. Recently, a study using PacBio HiFi to assemble the genomes of 32 accessions showed that SVs can play an important role in local adaptation^46^.

In this Article, we de novo assembled 69 reference-quality *Arabidopsis* genomes either using PacBio HiFi or Oxford Nanopore long-read technologies, including accessions from Europe, the Middle East, Asia, Africa, Madeira and North America. With such a geographic spread, we aim to capture and describe most of the diversity in *Arabidopsis* genomes worldwide and constitute a comprehensive resource for future studies bridging phenotypes and genotypes.

## Results

### Genomic relationship of 72 *A. thaliana* accessions

**Fig. 1:**
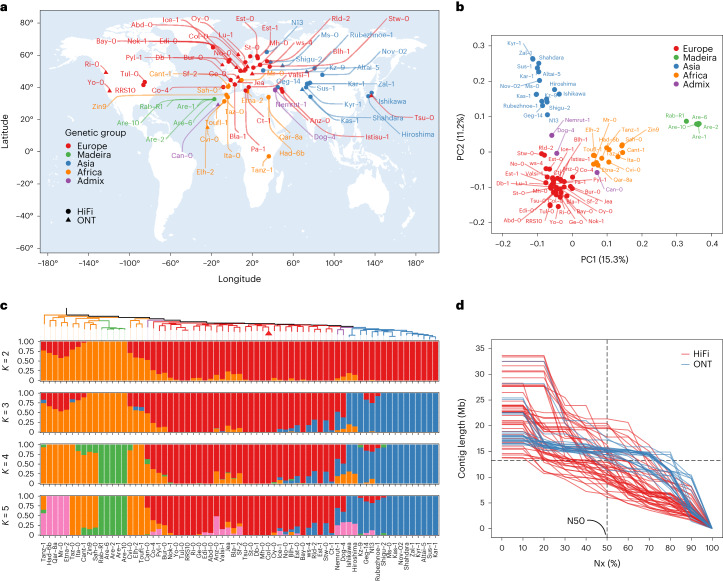
Geographic distribution and population analysis of 72 *A. thaliana* accessions. **a**, The geographic distribution of the 72 accessions in this study. The different sequencing technologies are indicated by differently shaped dots. **b**, A principal component analysis of the 72 accessions. Colored dots indicate the genetic classification of each accession; three accessions (Nemrut-1, Dog-4 and Can-0) were found to be admixed and are marked as purple dots. **c**, The phylogenetic tree and population structure of the 72 accessions with different numbers of ancestral kinships (*K* = 2, 3, 4 and 5). Each color represents one group. Each accession is represented by a vertical bar, and the length of each colored segment in each vertical bar represents the proportion contributed by ancestral populations. **d**, Assembly contiguity shown as the Nx (the length of the shortest contig that longer and equal length contigs represent x% of the assembly) plot of 69 accessions. Accessions sequenced by PacBio HiFi and Oxford Nanopore are colored in red and blue, respectively. The world map was generated in the ggplot2 package. Source data are provided as a source data file. Source data

**Extended Data Fig. 1:**
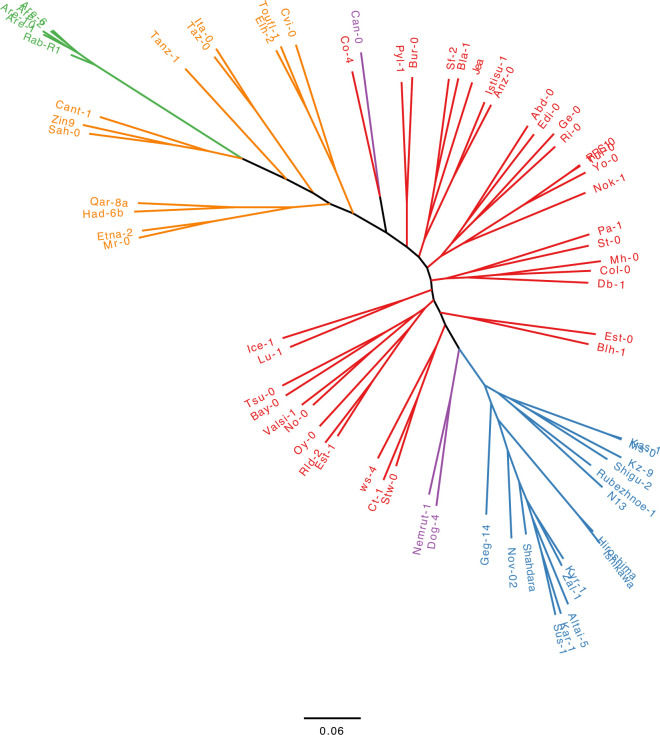
Phylogenetic tree based on SNPs in the 72 *A. thaliana* genomes. Tree branches (accessions) are coloured according to the genetic classification. Europe (red), Asia (blue), Madeira (green), Africa (orange) and admixture (purple).

We selected 72 *Arabidopsis* accessions across the global species distribution (Fig. 1a and Supplementary Table 1) and examined their genetic diversity and relationships through SNP analysis. Principal component, phylogenetic tree and admixture analyses showed that global genetic relationships between the 72 genomes broadly mirrored their geographic origins, as previously shown in this species^35^ (Fig. 1a,b). We identified four major genetic groups and named them after the geographic origin of the majority of their members: ‘Europe’ (35 accessions, including 4 accessions from recently colonized North America^47^), ‘Africa’ (13 accessions, including accessions from the Mediterranean rim), ‘Madeira’ (5 accessions exclusively from Madeira) and ‘Asia’ (16 accessions distributed from Eastern Europe to Japan). In addition, we identified three accessions (Nemrut-1, Dog-4 both from Turkey and Can-0 from Spain) that did not cluster in distinct groups but were labeled ‘admixed’ and originated from geographic regions between the distinct groups (Fig. 1a–c and Extended Data Fig. 1). We also found that Tsu-0, which is labeled as an accession from Japan, clustered together with the European accessions. This is consistent with previous reports^48,49^ suggesting that Tsu-0 was mislabeled, and we treated it as belonging to the ‘Europe’ genetic group from thereon.

To infer the evolutionary relationships among *A. thaliana* accessions, we constructed a species tree of the accessions (see below for further detail in pan-genome analysis, Supplementary Fig. 1), which confirmed their evolutionary relationships and was consistent with previous reports highlighting the African populations as the most divergent and probably most ancient lineages^36^.

### Chromosome-level assemblies of 69 *A. thaliana* accessions

**Extended Data Fig. 2:**
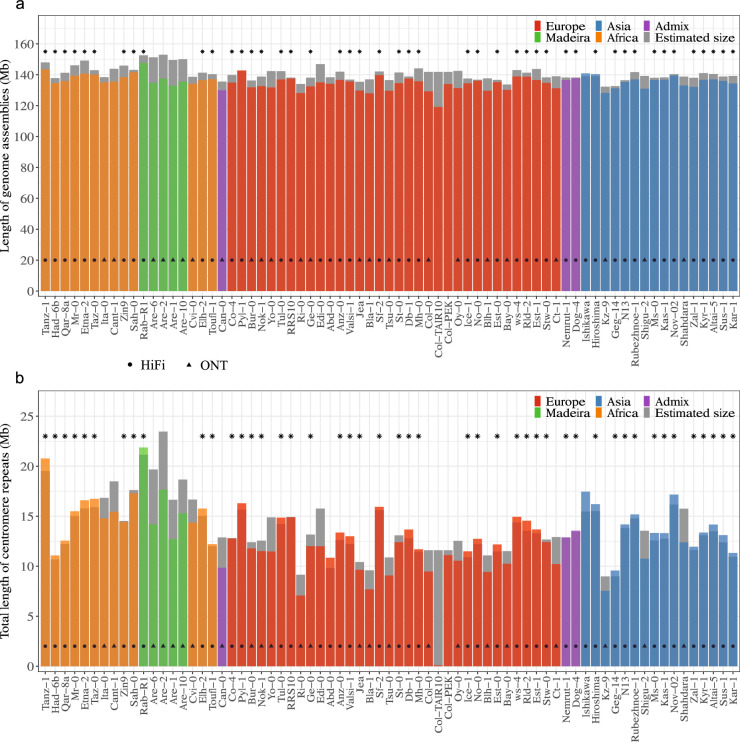
Comparison of the assembly and estimated lengths of genomes and centromeres. (**a**) Comparison of the assembly and genome sizes of the 69 accessions. The genome sizes were estimated based on k-mer from Illumina reads. (**b**) Comparison of the assembly and centromere sizes of the 69 accessions. The centromere sizes were estimated based on sequencing depths of Illumina reads. The estimated sizes are shown by grey bars. The assembly size of each accession are coloured according to its genetic classification. The most complete 46 genomes are marked by stars.

We generated genome assemblies for each of the 72 accessions using a combination of long-read (48 accessions with PacBio HiFi with a mean depth of 45×, and 24 accessions with Oxford Nanopore with a mean depth of 67×) and short-read sequencing, reference-guided scaffolding and manual curation (Methods). The quality of the assemblies was analyzed in six different aspects across the assemblies (Supplementary Tables 2–7, Extended Data Fig. 2 and Supplementary Figs. 2 and 3). Sixty-nine accessions were confirmed to be inbred lines, but Lu-1, Pa-1 and Istisu-1 showed signs of heterozygosity and thus were removed from the subsequent analysis (Methods). The remaining contig assemblies featured N50 values from 6.1 to 21.3 Mb with a mean of 13.3 Mb, and were scaffolded to the chromosome level (Fig. 1d and Supplementary Table 4).

**Fig. 2:**
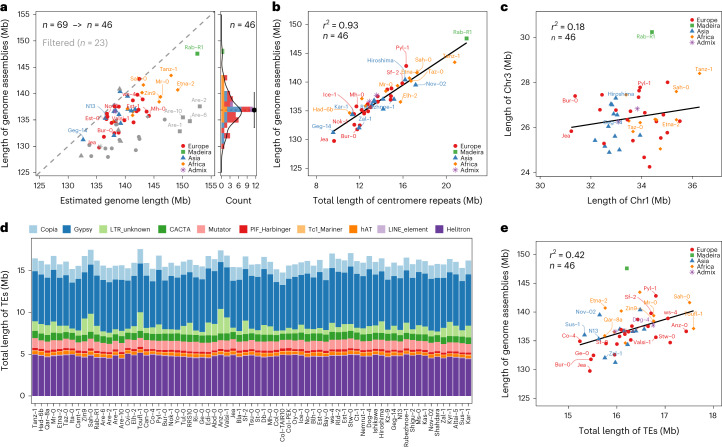
De novo assembly and annotation of 69 *A. thaliana* accessions. **a**, An assessment of the completeness of the 69 genome assemblies based on the comparison of estimated genome sizes and assembly features. The genome assemblies of 23 accessions were filtered out for genome size analyses based on the completeness ratio of genome and centromere assembly. **b**, A correlation analysis of genome assembly sizes and centromeric repeats. **c**, An example of a correlation analysis of assembly sizes of individual centromeres. **d**, The total length and composition of repetitive sequences of the genome assembly. **e**, A correlation analysis between the total lengths of genome assemblies and the TEs. Accessions are colored according to their genetic classification. Source data are provided as a source data file. Source data

The assembly sizes of the 69 accessions ranged from 128 to 148 Mb, with an average length of 135 Mb (Fig. 2a). Previous estimates of genome size variation in *A. thaliana* using flow cytometry were generally higher, ranging from 161 Mb to 184 Mb (ref. ^50^) (180 lines from Sweden, and the estimation for Col-0 was 166 Mb). However, genome size estimations based on flow cytometry are known to suffer from overestimation^51^. More recent estimates derived from *k*-mer analyses based on short read resequencing data of 89 accessions, indicated a range from 138 Mb to 175 Mb (ref. ^51^). This variation in genome sizes was found to be largely determined by 45S ribosomal DNA (rDNA) copy number variation^50^. We also used *k*-mers to estimate the genome sizes of our 69 accessions, and compared them to their assembly sizes (Fig. 2a). Some of the ONT read-based assemblies were substantially shorter than their estimated genome sizes, as their rDNA arrays and centromeres were not fully assembled (Fig. 2a and Extended Data Fig. 2).

With the aim of deciphering the underlying genomic features contributing to the variation in genome size, we selected the 46 most complete assemblies (42 accessions assembled with HiFi, and 4 accessions, Bur-0, Ge-0, Jea and Nok-1, assembled with ONT) based on both (i) the ratio of assembly and *k*-mer-based genome size estimation and (ii) the ratio of centromere repeat length and read coverage-based centromere size estimation (Fig. 2a and Extended Data Fig. 2). The assembly sizes of these accessions ranged from 130 to 148 Mb. Their centromeric repeat arrays were on average 14 Mb long (across all five chromosomes) ranging from 10 to 22 Mb and were highly correlated with assembly sizes (Pearson’s correlation *r* = 0.93, *P* < 2.2 × 10^−16^, Fig. 2b, Extended Data Fig. 2 and Supplementary Table 7). This showed that the variation of the size of the centromeric arrays in *Arabidopsis*, as recently described by Wlodzimierz et al.^52^, is a major contributor to the variation of genome size. Transposable elements (TEs) were annotated with a pan-TE library generated from initial TE annotations of the individual assemblies. The size of TE space (that is, genomic regions with similarity to TEs) was surprisingly similar between the genomes, with a mean length of 16.1 Mb, ranging from 15.2 to 17.6 Mb (Fig. 2d,e). Among them, long terminal repeat (LTR) retrotransposons (Copia, Gypsy and LTR unknown) and Helitrons made up the largest TE fractions and constituted 6.4% and 3.5% of the genome, respectively (Fig. 2d). Accordingly, the variation in the size of TE space between the accessions was moderately correlated with the total assembly size (Pearson’s correlation *r* = 0.42, *P* = 0.003, Fig. 2e). This suggests that genome size variation in *Arabidopsis* (excluding the variation of 45S rDNA size) is mostly dominated by centromeric repeat length and that TEs are only minor contributors. This is in sharp contrast to the situation between plant species, where the main determinants of genome size variation are ploidy levels and TE content^53^. In species with high TE content, such as rice^23^, TE space variation can contribute more largely to intraspecific variation in genome size. Interestingly, however, even though cumulative centromere size determines genome size in *A. thaliana*, the sizes of individual chromosomes were only weakly or not correlated in size (Pearson’s correlation *r* = 0.2, *P* = 0.171, Fig. 2c and Supplementary Figs. 3 and 4), indicating that the sizes of individual centromeres evolve independently from each other.

### A quasi-fixed karyotype across the *A. thaliana* species range

**Fig. 3:**
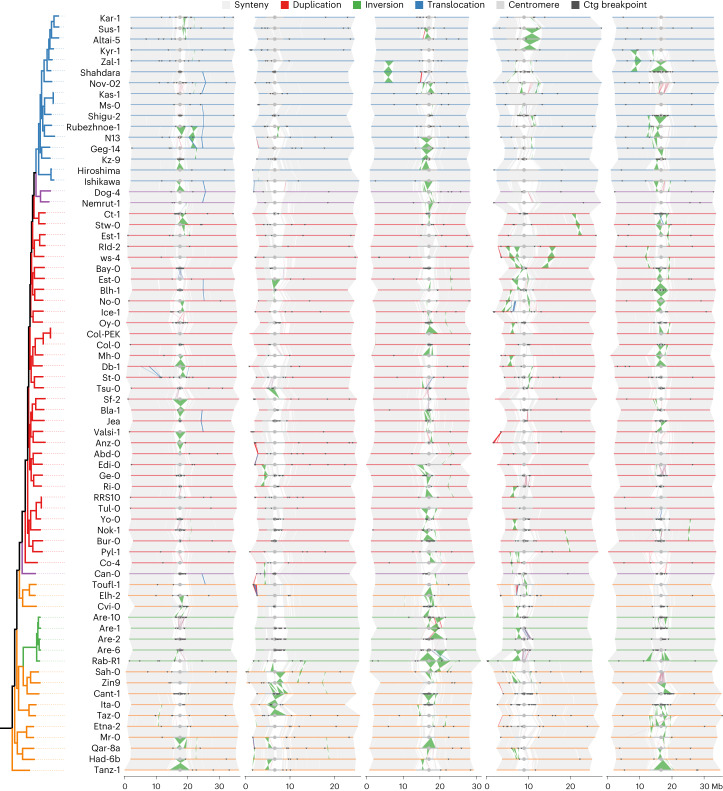
Whole-genome alignments of 69 *A. thaliana* accessions. The chromosomes of each accession were represented by segments, which are colored in line with the genetic classification. The gray segments represent the centromeric regions, and the middle points are indicated by gray circles. The syntenic regions between chromosomes are shown in light gray. The structural rearrangements, including duplications, inversions and translocations, are colored red, green and blue, respectively. Contig (ctg) breakpoints are marked by black triangles. Source data are provided as a source data file. Source data

**Extended Data Fig. 3:**
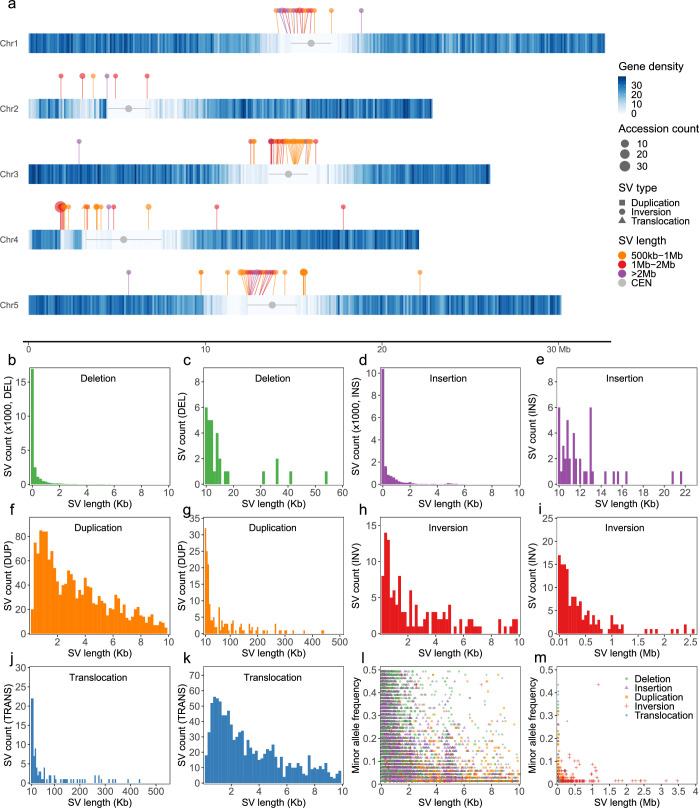
The distribution of length and minor allele frequency of SVs in the 69 genomes. (**a**) The chromosomal distribution of SVs ( > 500 kb) in the 69 genomes along chromosomes of Col-PEK. The heatmap indicates the gene density. The centromeres are indicated by grey circles and segments. The colour, shape and size of the individual points represent the size, type and allele frequency of the SVs. (b-k) Length distributions of SVs (deletions, insertions, duplications, inversions and translocations) with size from 20 bp to 10 kb and longer than 10 kb, separately. (l-m) The minor allele distribution of SVs with sizes from 20 bp to 10 kb and longer than 10 kb, separately. The colour and shape of points represent the type of the SVs.

**Extended Data Fig. 4:**
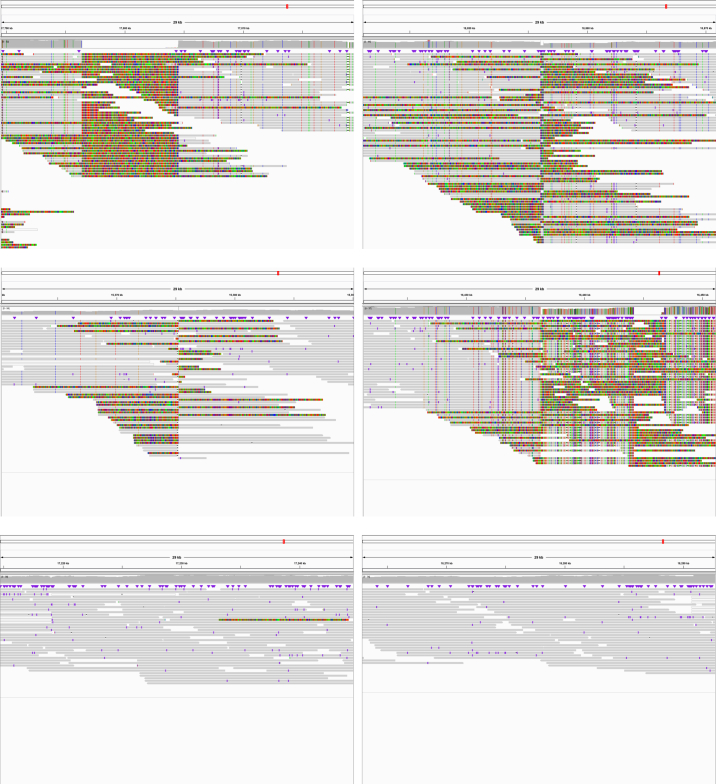
Alignment of long-reads in the breakpoint regions of the inversion in Stw-0. The top, middle and bottom panels show the alignment of long-reads from Stw-0 against Col-PEK, the closest accession Ct-1, and the Stw-0 assemblies, with a window of 30 kb covering the left and right breakpoints of the detected inversion, respectively.

Chromosome-level genome assemblies allow accurate analysis of large-scale genomic rearrangements and genome colinearity^13^. Using pairwise whole-genome alignments, we found that the chromosome arms hardly contained any major rearrangements and were highly syntenic across all genomes, even when comparing genomes from distant parts of the world (Fig. 3, Supplementary Figs. 5–9 and Extended Data Fig. 3). Large insertion/deletion polymorphisms are absent from chromosome arms, the vast majority being smaller than 20 kb and the largest reaching ~55 kb (Extended Data Fig. 3). Inversions along chromosome arms are also rare, but are larger than insertions/deletions, with a few cases above one megabase. We identified a total of seven inversions on chromosome arms, almost all present in single accessions (Fig. 3, Supplementary Figs. 5–9 and Extended Data Fig. 3): a ~2.4 Mb inversion on chromosome 3 in Shahdara, a ~2.3 Mb inversion on chromosome 5 in Zal-1, a ~2.2 Mb inversion on chromosome 1 in N13, a ~1.8 Mb inversion on chromosome 4 in Ws-4, a ~1.2 Mb inversion on chromosome 4 in Stw-0 and a ~1 Mb inversion on chromosome 2 in Ge-0 (validated by long read alignment, Extended Data Fig. 4 and Supplementary Figs. 10–14). A notable exception to this was the well-described ~1.2 Mb inversion on chromosome 4 (refs. ^5,41^), which was observed in eight accessions (including Col-0) and which partially overlapped with the heterochromatic, pericentromeric regions. This inversion is found in the ‘Europe’ genetic group among geographically distant accessions (for example, Yo-0 from North America and Ws-4 from Belarus), thereby suggesting a long-lived segregation of this particular inversion.

The karyotype of *A. thaliana* is quite different to the estimated ancestral karyotype of the Brassicaceae, which is still conserved in the sister species *Arabidopsis lyrata*^54,55^ and involved a species-specific deletion of three functional centromeres along with major chromosomal rearrangements and fusions^56^. The high structural similarity between the 69 genomes implied that the derived karyotype of *A. thaliana* arose during or shortly after speciation and was maintained virtually unchanged during the global spread of this species that colonized contrasted ecological habitats.

In contrast to the chromosome arms, large rearrangements of different types were highly abundant in and near the centromeres and as a result led to numerous different centromeric haplotypes (Fig. 3, Supplementary Figs. 5–9 and Extended Data Figs. 3 and 5).

**Fig. 4:**
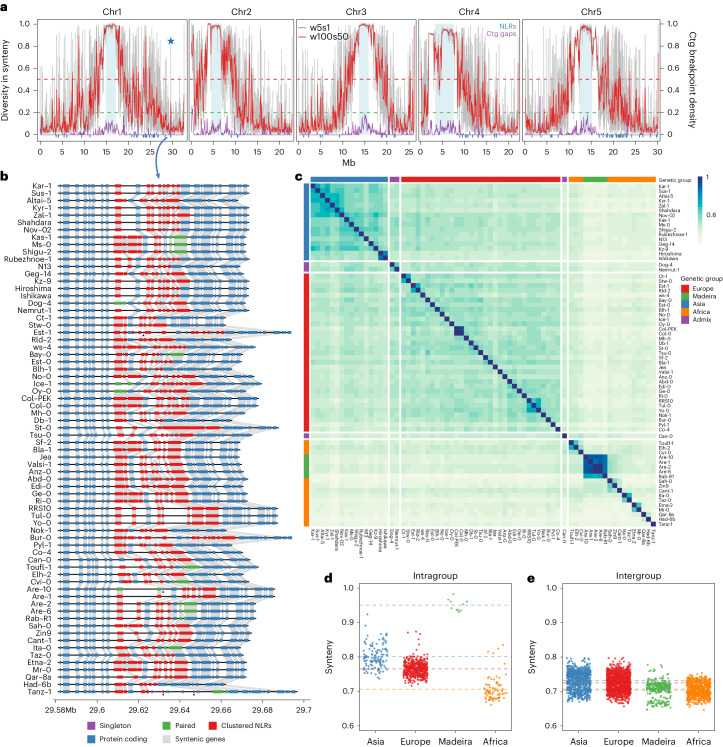
Characterization of the synteny landscape of 69 *A. thaliana* accessions. **a**, The diversity in synteny and density of contig (ctg) breakpoints along the five chromosomes. The diversity of synteny is shown at two resolutions, in red and gray (sliding window: 100 kb window size with 50 kb step size in red; 5 kb window size with 1 kb step size in gray). The density of contig breakpoints is shown in purple (100 kb window size with 50 kb step size). The blue bar indicates NLR genes. The horizontal dashed green and red lines indicate thresholds for synteny diversity values of 0.25 and 0.50. Mb, megabases. **b**, The local syntenic gene order in a highly divergent region of chromosome 1. The protein-coding genes and NLRs (singleton, pair and cluster) are presented by blue, purple, green and red rectangles. The gray links between the rectangles indicate homologous relationships. The private genes were marked by black triangles. **c**, Pairwise synteny relationships measurement along chromosome arms. **d**,**e**, A comparison of synteny relationships within (**d**) and between (**e**) groups. Source data are provided as a source data file. Source data

To quantify colinearity with higher resolution, we measured colinearity in sliding windows along the chromosomes and calculated average pairwise diversity in synteny of each of the newly assembled genomes against a recent genome assembly of Col-0 (ref. ^57^) (Col-PEK), which includes most of the centromeric regions (Fig. 4a). Average pairwise diversity in synteny ranges from 0 to 1, with 1 denoting complete absence of colinearity between the genome of a group and 0 indicating colinearity among all the genomes. Overall, for the 69 genomes, around 50% of the genome was highly colinear, with an average pairwise diversity in synteny lower than 0.2, which was exclusive to the chromosome arms (Fig. 4a and Supplementary Fig. 15). In contrast, ~33% of the genome was highly diverse with an average pairwise diversity in synteny of over 0.5, mostly including regions in and near the centromeres (Fig. 4a and Supplementary Fig. 15). We observed transitions between regions with very high and very low synteny in the peri-centromeres, which covered several Mb around each of the centromeres.

The broad colinearity in the chromosome arms showed a few interesting exceptions. The short arms of chromosomes 2 and 4, where rDNA clusters and nucleolus organizer regions are located, showed high levels of rearrangements, like the lower arms of chromosome 1 (~25 Mb) and 5 (~20 Mb) where large and highly diverse resistance *R* gene clusters reside (Fig. 4a)^58^. Also, the ~1.2 Mb inversion on chromosome 4 was marked by high diversity in synteny consistent with the fact that it segregates in several groups (Fig. 4a). In addition, we found individual spikes of high structural diversity in regions that were otherwise highly colinear. As previously described, such local hotspots of rearrangements were enriched for *R* gene clusters^3^. For example, at ~30 Mb on chromosome 1, synteny was broken by the high and variable copy number changes in a nucleotide-binding and leucine-rich repeat (NLR) gene cluster (Fig. 4b).

In addition, pairwise genome-wide colinear relationships reflected the genetic and geographical groups (Fig. 4c–e and Supplementary Fig. 16), implying that structural differences can recapitulate our genetic grouping based on SNPs (Extended Data Fig. 1 and Supplementary Fig. 17), which was also recently shown with read alignment-based SV calls^38^. Small subgroups of accessions exhibited increased colinearity. These corresponded to geographical clusters, including the Japanese accessions (Hiroshima and Ishikawa), the North American accessions (Yo-0, RRS10 and Tul-0) and the Madeiran accessions (Are-1, Are-2, Are-6, Are-10 and Rab-R1). Interestingly, we also found that the colinearity among African accessions was lower than the colinearity between European or Asian accessions (Fig. 4c–e), probably reflecting the higher genomic diversity in Africa^36^.

### The pan-genome of *A. thaliana*

**Fig. 5:**
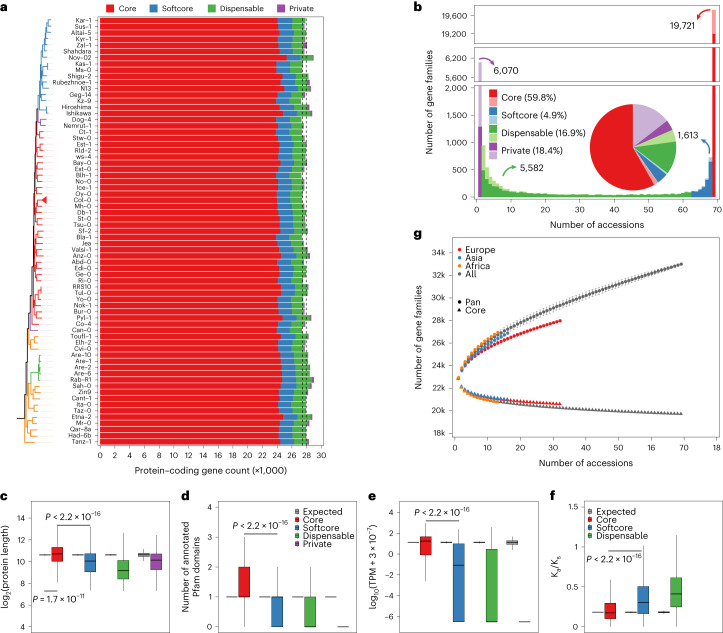
Pan-genome analysis of 69 *A. thaliana* accessions. **a**, The annotated protein-coding genes, and composition of core (red), softcore (blue), dispensable (green), and private (purple) genes in each individual accession. **b**, The number and proportion of core (red), softcore (blue), dispensable (green) and private (purple) gene families in the pan-genome. The split–merge cases are indicated by light colors. **c**–**f**, The protein length (**c**), number of annotated Pfam domains (**d**), gene expression landscape (**e**) and K_a_/K_s_ (**f**) of core (red), softcore (blue), dispensable (green) and private (purple) genes of Col-0. The expression level of the gene is defined as the median value in the 79 organs and developmental stages. The expected dataset was made by 1,000 simulations with the same data size of the testing dataset. The two-sided Mann–Whitney test was performed, and the *P* value is indicated respectively. Intervals for boxplots: center, median (50th percentile); lower bounds of box, 25th percentile (Q1); upper bounds of box, 75th percentile (Q3); lower whisker, maximum of (minima, Q1 − 1.5 × IQR); upper whisker, minimum of (maxima, Q3 + 1.5 × IQR). IQR, interquartile range (range of Q1 to Q3). **g**, The increase of the pan-genome size and the decrease of core-genome size in the whole population (gray), Europe (red), Asia (blue) and Africa (orange) groups. Accessions were sampled as 2,000 random combinations of each given number of accessions ranging from 2 to 67. The mean number of gene families is shown with the standard deviation. Source data are provided as a source data file. Source data

We annotated 27,246 to 28,989 protein-coding genes in each of the 69 assemblies (Fig. 5a), compared to 27,445 genes in the reference sequence^59^. To unravel the gene repertoire of *A. thaliana*, we clustered all 1,928,005 genes combined with the gene sets of the reference sequence (Col-0 and Araport11), an additional recent telomere-to-telomere (T2T) Col-0 assembly (Col-PEK) and the reference sequences of the sister species *A. lyrata* and *Capsella rubella*, which served as outgroups. In total, we identified 36,991 gene families across the 73 genomes, including 13,328 single-copy gene families that were used for constructing the phylogeny of the *A. thaliana* accessions (Supplementary Fig. 1).

**Extended Data Fig. 6:**
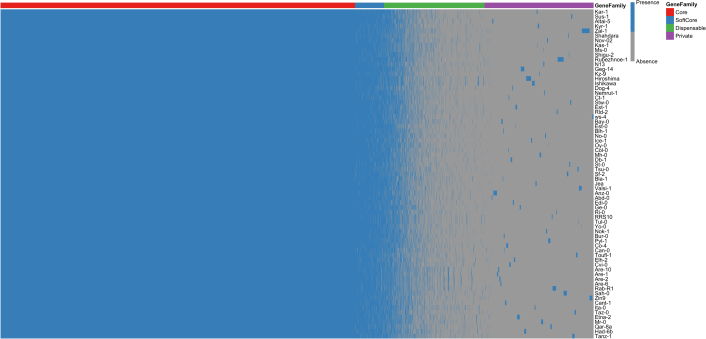
Presence and absence of pan gene families in the 69 *A. thaliana* genomes. Each column indicates a non-redundant gene (gene family), and each row indicates an accession. The gene families were grouped by their categories including core, softcore, dispensable and private. Accessions are ordered according to their genetic relationship.

Excluding the genes from the reference genome, the T2T Col-0 accession, *A. lyrata* and *C. rubella*, we found that 32,986 gene families included genes from at least one of the 69 *A. thaliana* genomes. Of those, 19,721 (60%) were present in all 69 genomes and were defined as core gene families. Among the gene families that appear nonessential, 1,613 (5% of total) were present in 63–69 accessions (>90% of the accessions), defined as softcore; 5,582 (17%) were present in 2–62 genomes, defined as dispensable; and the remaining 6,070 (18%) gene families were present in only one genome, defined as private gene families (Fig. 5b and Extended Data Fig. 6). On average, a single genome consisted of 86.8% core, 6.7% softcore, 6.1% dispensable and 0.4% private genes (Fig. 5a). As expected, the increased size of our collection reduced the number of core gene families as compared to a previous estimation, which was based on eight assemblies only^3^. However, our core gene family number estimate was similar to one of a recent study where 21,545 gene families were annotated as core gene families among 32 accessions^46^, indicating that our estimation probably reflects the actual core gene set of *A. thaliana*. In contrast, however, we found a much higher number of private genes in the genomes (18.4% of all gene families) as compared to the previous study. To understand the origin of the private genes, we explored local sequence homology and structure of all gene families, and found that 6,954 gene families were formed by differences in annotation, where gene models are split or merged differently in association with local polymorphisms (Methods). The split–merge cases represent 2.7%, 11%, 26.2% and 78.9% of the core, softcore, dispensable and private gene families, respectively (Fig. 5b, pastel colors). Even though these gene families could result from errors in the annotation, split–merge cases may also represent differences in the transcriptomes with functional consequences. For all the remaining 1,281 private gene families, no sequence similarity can be detected in the other accessions, suggesting that they represent de novo evolved genes.

We found that the length of the encoded protein sequences of the core genes was longer and more often matched Pfam domains as compared to other types of gene (Mann–Whitney test between core and softcore genes, *P* < 2.2 × 10^−16^, Fig. 5c-d). Gene expression across 79 organs and developmental stages (measured in Col-0 (median ≥1 transcript per million (TPM))), revealed a much higher fraction of expressed genes with significantly higher expression levels in the core and softcore gene families as compared to the private genes (even though 30.8% of the Col-0 dispensable genes (488/1,584) and 10% of the Col-0 private genes (3/31) were still expressed) (Mann–Whitney test between core and softcore genes, *P* < 2.2 × 10^−16^, Fig. 5e). Moreover, core genes showed significantly lower nonsynonymous/synonymous substitution ratios (K_a_/K_s_) than accessory genes, suggesting that core genes were more functionally constrained than accessory genes (Mann–Whitney test between core and softcore genes, *P* < 2.2 × 10^−16^, Fig. 5f). This is corroborated by the functional categories of the different types of gene family that were analyzed with a Gene Ontology (GO) enrichment analysis. Core genes were enriched for basic and essential biological processes, including metabolic, cellular, developmental processes, reproduction and regulation of biological process (Supplementary Fig. 18 and Supplementary Table 8). Among them, the 27 meiosis-specific genes^60^ were all highly conserved, and were present in the same copy numbers (26 in one copy, and 1 in two copies) across the 69 accessions (Supplementary Table 9). Accessory genes (softcore, dispensable and private categories, analyzed independently or together) were enriched for biological processes such as cell killing and defense response (Supplementary Fig. 19 and Supplementary Table 10). Altogether, while this suggests that the accessory genes are substantially different from the core genes, it also suggests that a fraction of those have functional features and could contribute to phenotypic diversity and adaptation.

Finally, we measured the sizes of the pan-gene sets of the 69 *A. thaliana* accessions by subsetting the genomes (Fig. 5g and Supplementary Fig. 20). Even after adding all genomes, the pan-genome gene set did not reach a plateau, indicating that our set of accessions did not capture the entire complement of diverse gene families in this species. The reason for this was the high number of gene families that were only present in one or a few of the accessions (‘dispensable’ and ‘private’ gene families), hinting at an untapped genetic diversity.

## Discussion

In this study, we have generated 69 reference-quality genome assemblies, which capture a large degree of the genetic diversity in *A. thaliana*. These assemblies were generated from accessions selected from Central Africa to Iceland and from North America to Japan, but despite these huge geographical distances, genome structure was highly conserved among plants. The assemblies also revealed a total of 10,420 novel protein-coding gene clusters, which are absent from the reference genome (Col-0 and Araport11) and provide a very powerful resource to study the genetic basis of hitherto undescribed variation. In addition, our collection of genome assemblies contains the parental strains of powerful and publicly available material such as recombinant inbred lines^61,62^. Genome assemblies will help to unravel the genetic basis of important traits relying on complex structural variations^63–66^. Finally, these 69 genomes, together with others, provide a great resource to study the mechanisms of genome dynamics, including recombination. These resources pave the way for further functional genomic investigation.

## Methods

### Plant material and whole-genome sequencing

We ordered the seeds of 66 accessions from the Versailles Arabidopsis Stock Center at Jean-Pierre Bourgin Institute and received the seeds of the remaining 6 accessions (Elh-2, Ice-1, Rab-R1, Tanz-1, Taz-0 and Zin9) from Angela Hancock (MPIPZ). The accession numbers of seeds from Versailles are presented in Supplementary Table 1. Plants were grown in greenhouses or growth chambers.

For the 48 accessions that were sequenced with PacBio HiFi, high-molecular-weight (HMW) DNA was prepared from a pool of 30–40 4-week-old plants using the NucleoBond HMW DNA kit. The HiFi libraries were prepared using the SMRTbell prep kit 3.0 and the BluePippin cartridge for enriching fragments greater than 9 kb up to 50 kb. Finally, HiFi sequencing was performed on the Sequel IIe platform at the Max Planck Genome-centre Cologne. SMRTlink software (PacBio) was used to demultiplex and extract HiFi datasets. DNA form a single plant leave was used to prepare PCR-free short-read libraries according to the protocol of the NEBNext Ultra II DNA PCR-free Library Prep Kit for Illumina (New England Biolabs). Libraries were then sequenced with 2 × 150 paired-end reads on the NextSeq 2000 platform.

As previously described^67^, for the 24 accessions that were sequenced with Oxford Nanopore, HMW DNA was extracted from 3-week-old plants according to the protocol described by Russo et al.^68^. The subsequent library preparation and sequencing were performed at the GeT-PlaGe core facility, INRAE Toulouse. ONT libraries were prepared using the EXP-NBD103 and SQK-LSK109 kits according to the manufacturer’s instructions and using 4 µg of 40 kb-sheared DNA (Megaruptor, Diagenode) as input. Pools of six samples were sequenced on one R9.4.1 flowcell. Between 14 and 20 fmol of library was loaded on each flowcell and sequenced on a PromethION instrument. Illumina libraries were prepared using the Illumina TruSeq Nano DNA HT Library Prep Kit. Libraries were then sequenced with 2 × 150 bp paired-end reads on the Hiseq3000 platform.

### De novo genome assembly

Genome heterozygosity and size were estimated using Jellyfish v2.2.6 (ref. ^69^) and findGSE^51^.

For the 48 accessions that were sequenced with PacBio HiFi, the initial de novo assembly was performed using three different assembly tools: Canu v2.1.1 (ref. ^70^), Flye v2.7 (ref. ^71^) and Hifiasm v0.16.1 (ref. ^72^). Then, purge_dups v1.2.5 (ref. ^73^) was used to purge haplotigs and overlaps in the assemblies from Canu and Hifiasm. To improve contiguity, we combined the assemblies derived from the three assemblers using quickmerge v0.3 (ref. ^74^). First, the assembly with the best quality (longest contiguity measured as N50, assembly size and correctness, centromere coverage and so on) was selected as query, and the assembly with second longest N50 was used as reference to join contigs in the query assembly (Supplementary Table 2). The resulting assembly was further improved by using the third assembly as reference. Then, we used a homology-based scaffolding tool, RaGOO v1.1 (ref. ^75^), to order and orient contigs on the basis of whole-genome alignments to the Col-CEN genome^76^. Manual evaluation and correction were performed based on the whole-genome alignment and position of centromeric repeats. Finally, to close the gaps in scaffolds, we ran four rounds of MaSuRCA v4.1.0 (ref. ^77^) by using the HiFi reads and the three assemblies individually.

For the 24 accessions that were sequenced with Oxford Nanopore, the long reads were filtered for adapters, short (<1 kb) or low-quality reads (mean quality >70) using Porechop v0.2.4 (https://github.com/rrwick/Porechop) and Filtlong v0.2.1 (https://github.com/rrwick/Filtlong). De novo assembly of each genome was initially performed using SMARTdenovo (https://github.com/ruanjue/smartdenovo)^78^ and Flye. To fix base errors in the initial assemblies, we polished the genome by running three rounds of Racon v1.4.10 (ref. ^79^) with long reads, and four rounds of NextPolish v1.3.1 (ref. ^80^) with short reads. Then, assemblies were purged using purge_dups. Similar as for the process described above for HiFi datasets, assemblies were further improved, corrected and scaffolded (Supplementary Table 3). Finally, scaffolds were polished using NextPolish.

### Genome assembly evaluation

To evaluate the completeness of each genome assembly, compleasm v0.2.2 (ref. ^81^) was used with the OrthoDB database brassicales_odb10 (brassicales, 2020-08-05). We evaluated the consensus quality value (QV) and completeness of genome assemblies on the basis of the *k*-mers spectrum of Illumina whole-genome sequencing reads, using Merqury v1.3 (ref. ^82^) with default parameters, to estimate the goodness and completeness of the reference protein-coding genes (TAIR10 and Araport11) in each genome assembly. The reference genes were aligned against the genome assemblies using blastn^83^, and reference genes were considered well assembled in genome assemblies which were aligned with identity ≥80 and coverage ≥0.9. We also used Liftoff v1.6.3 (ref. ^84^) to ‘lift over’ the reference genes to the 69 genome assemblies, with parameters ‘-copies -sc 0.90 -polish’. Additionally, to evaluate the assembly continuity, LTR Assembly Index (LAI) was calculated for each genome assembly using LTR_retriever v2.9.0 (ref. ^85^).

Centromeric and telomeric repeats were annotated using Bowtie2 v2.4.4 (ref. ^86^) (-a–very-sensitive) to search for the consensus sequence of the 178-bp and 7-bp repeat motifs in each genome assembly, respectively. To estimate the copy number and length of centromeric repeats, we compared the sequencing depths obtained from aligning Illumina short reads against the genome assembly and concatenated sequence of four copies of the repeat motif, using Bowtie2 (-k 1), separately. The sequencing depth was calculated using samtools v1.9 (ref. ^87^) with the parameter setting ‘-Q 1 -d 0 -a’.

We evaluated the assembly quality in the following six aspects, and found that (1) the assembled genome size is comparable to the length of T2T assembly of Col-0 reported by recent studies^57,76,88^ (Fig. 2a, Supplementary Figs. 3 and 21, and Supplementary Table 4); (2) the completeness estimated by Benchmarking Universal Single-Copy Orthologs was 99.8%, which is comparable to the Col-0 reference and T2T genomes (Supplementary Fig. 2 and Supplementary Table 4); (3) on the basis of the *k*-mer-based estimation, the assembled genomes showed a mean of 53.4 QV and 98.5% completeness (Extended Data Fig. 2 and Supplementary Table 4); (4) the analysis of completeness of reference protein-coding genes by homology search against the assembled genomes (BLASTP and Liftoff), a mean of 97.3% and 98.4% were successfully assembled (Supplementary Table 5); (5) on the basis of the LAI (mean of 22), which reached the ‘gold standard’ level (LAI >20) (Supplementary Table 6); (6) on the basis of the completeness of centromere repeats (mean of 96%), estimated using the Illumina short-read dataset (Extended Data Fig. 2 and Supplementary Table 7). Three accessions, Lu-1, Pa-1 and Istisu-1, had lower values in the measurement of QV and *k*-mer completeness. Further alternative allele frequency analysis indicated the presence of heterozygosity, which are unexpected in a pure line, probably due to the mixture of two distinct lineages or segregation of polymorphism in the population. These three accessions were ignored in subsequent analyses (Supplemental Fig. 28). These results suggested that the quality of all 69 genome assemblies was comparable to that achieved by the Col-0 reference and T2T genome assemblies, indicating high continuity and completeness.

### Annotation of repetitive elements

To annotate the TEs in the 69 genome assemblies, we first generated a nonredundant TE library for each accession, using the Extensive de novo TE Annotator (EDTA) v2.0.1 (ref. ^89^) with parameters ‘–overwrite 1–sensitive 1–anno 1–evaluate 1’. Then, all the individual TE libraries were combined to construct a pan-TE library using panEDTA^90^. RepeatMasker v4.1.1 (http://www.repeatmasker.org) was further employed to re-annotate the repeat regions with parameters ‘-q -div 40 -cutoff 225’ and the pan-TE library.

### Gene prediction and annotation

Protein-coding genes were annotated on the basis of a strategy that integrated ab initio gene prediction, transcriptome-based de novo transcript assembly and homologous protein sequence alignment. First, four ab initio gene prediction tools were used: Augustus v3.3.3 (ref. ^91^), GeneMark v4.62 (ref. ^92^), GlimmerHMM v3.0.4 (ref. ^93^) and SNAP (version 2006-07-28)^94^. Second, we collected a list of 308 public RNA sequencing (RNA-seq) datasets for 28 accessions from the NCBI SRA database (Supplementary Table 11). The quality of short reads was checked with FastQC. Trimmomatic v0.39 (ref. ^95^) was used to remove potential adapter and low-quality sequences, with parameters ‘LEADING:3 TRAILING:3 SLIDINGWINDOW:4:15 MINLEN:36’ for single-end reads and ‘LEADING:3 TRAILING:3 SLIDINGWINDOW:4:15 MINLEN:36’ for paired-end reads. To obtain the protein sequence of the transcript, the reads were then processed with Trinity v2.14.0 (ref. ^96^) to assembly transcript sequences, and TransDecoder v5.5.0 (https://github.com/TransDecoder/TransDecoder) to identify candidate coding regions. The predicted longest open reading frames were searched against the UniPort database^97^ using DIAMOND v2.0.4 (ref. ^98^) with the parameter setting ‘-k 1 -f 6 -e 1e-5–ultra-sensitive’, and Pfam database^99^ using hmmsearch from HMMER v3.1b2 (http://hmmer.org). Then, we merged all the predicted protein sequences to generate the pan-pep library and selected representative sequences using CD-HIT v4.6.8 (refs. ^100,101^) (-c 0.98). Third, protein sequences of *A. thaliana* (447_Araport11), *A. lyrata* (384_v2.1), *Oryza sativa* (323_v7.0) and *Solanum lycopersicum* (514_ITAG3.2), which were downloaded from Phytozome v13 database^102^ and the pan-pep library, were aligned to each genome assembly using Exonerate v2.2.0 (ref. ^103^) (–percent 70–minintron 10–maxintron 60000). Finally, all different evidences of gene models were integrated using EVidenceModeler v1.1.1 (ref. ^104^). The resulting gene models, especially for those from nonscaffold contigs, were further evaluated by comparing to the National Center for Biotechnology Information (NCBI) nonredundant (NR) database using DIAMOND, outside which Brassicaceae proteins were excluded.

Noncoding genes were annotated by integrating the predictions from Barrnap v0.9 (https://github.com/tseemann/barrnap), Infernal v1.1.4 (ref. ^105^) (Rfam database v14.8) and tRNAscan-SE v2.0.9 (ref. ^106^). Noncoding RNA and TE-related genes were identified by checking alignment/overlap between predicted gene models and TE sequences (TAIR10), representative gene models (Araport11), TE genes (Araport11), TE and noncoding RNA annotations of each assembly.

Disease resistance genes were identified by using NLR-Annotator v2.1 (ref. ^107^) and RGAugury^108^. NLRs have been reported to be mostly present in pairs or cluster^23,58^. Pair NLRs were defined as fewer than two non-NLR genes between the two NLRs. Cluster NLRs were defined as more than two NLRs with fewer than two non-NLR genes between any two NLRs. The remaining NLRs were labeled as singletons.

The resulting gene models were further annotated functionally using InterProScan v5.59-91.0 (ref. ^109^) (parameters: -f TSV -t p -iprlookup -goterms -pa). The GO enrichment analysis was performed using AgriGO v2.0 (ref. ^110^).

### Gene-based pan-genome construction and analysis

All the protein-coding genes from the 69 assembled genomes, representative protein-coding genes from Col-0 TAIR10 (refs. ^32,111^), Col-PEK^57^ and two out-species, *A. lyrata* (384_v2.1) and *C. rubella* (474_v1.1)^102^, were clustered using OrthoFinder v2.5.4 (ref. ^112^) with the parameter setting ‘-S diamond_ultra_sens’, resulting 37,921 gene clusters for 73 genomes. We used Liftoff to ‘lift over’ all the predicted protein-coding genes (including genes from the Col-0 TAIR10) to each of the 69 genome assemblies, with parameters ‘-copies -sc 0.90 -polish’. The gene locus, that is, low allele frequency, in each genome was checked for the presence of homologous genes (95% coverage for both query and hit genes, and reciprocal best hit) from the other accessions, and then, the related orthogroups were fused. Furthermore, the potential split–merge cases were also evaluated for the alignment and coverage between the query gene and representative gene (across the 69 accessions) or TAIR10 reference genes. After correction, we obtained 36,991 gene clusters for 73 genomes, and 32,986 gene clusters for the 69 assembled genomes. The orthologous groups were then classified into four categories: core gene clusters were defined as the genes shared between all the 69 genomes; softcore gene clusters were present in more than 90% of accessions (63–68); dispensable gene clusters were found in more than one accession (2–62); and private gene clusters that were accession specific. To estimate the pan-genome and core-genome size (the number of gene families defined by OrthoFinder), we carried out 2,000 random samplings of accessions for each number of sample size (ranging from 2 to 67) from the 69 accessions.

### Detection of SNPs and indels

The Illumina whole-genome sequencing short-reads of the 72 accessions (with mean depths of 43×) were aligned against the Col-PEK genome by BWA v0.7.15-r1140 with default parameters, and duplicated reads were removed using samtools. Then, SNPs and small indels (ranging from 1 to 20 bp) were detected and filtered for each genome assembly and merged by inGAP-family^113^. The resulting variants were further processed by VCFtools v0.1.16 (ref. ^114^) to obtain the high-quality and informative SNP list (parameters: ‘–maf 0.05–max-missing 0.2–min-alleles 2–max-alleles 2–min-meanDP 6–max-meanDP 226’). SNPs that were located in the region of centromeric repeats and TEs were removed. We found a total of 7,056,033 SNPs, among which 2,254,527 were common and located in noncentromeric and non-TE regions, with a density of one SNP per 59 bp.

### SV detection and analysis

To fully take advantage of the 69 high-contiguity genome assemblies, we performed the whole-genome alignment against the Col-PEK genome using minimap2 v2.21-r1071 (refs. ^115,116^) (parameters: -ax asm5–eqx), and SyRI v1.6 (ref. ^13^) was applied to identify SVs with default parameters. SVs (20 bp to 10 kb) from the 69 genome assemblies were merged by SURVIVOR with the parameters ‘1000 1 1 0 0 1’. SVs longer than 10 kb from 69 accessions detected by SyRI were retained and merged by SURVIVOR (parameters: ‘20000 1 1 0 0 1’).

### Population and phylogenetic analysis

For the SNPs in the 72 *A. thaliana* accessions, linkage pruning was performed by PLINK v1.90b6.18 (ref. ^117^), with the parameters ‘–indep-pairwise 50 10 0.1’. Then, principal component analysis (PCA) was conducted by PLINK, which showed that PC1 splits Madeira and Africa accessions from the North America–Europe–Asia group, while PC2 further divides Asia accessions from the others (Fig. 1b). We performed population structure inference using ADMIXTURE v1.3.0 (ref. ^118^), with the number of population settings ranging from 2 to 5. For *K* = 2, we found a division between Africa and the other accessions. When *K* = 4, we saw a new subgroup (Madeira) within the Africa group. When *K* = 5, the subgroup Sicily and Lebanon emerged within the Africa group (Fig. 1c).

To build the phylogenetic tree of the 69 accessions, a total of 13,328 single-copy orthologous gene clusters were selected and used for generating the amino acid alignment using MUSCLE v3.8.31 (ref. ^119^) with default parameters. For the amino acid alignment of each ortholog group, the nucleotide sequence that corresponds with the amino acid sequence was extracted by seqkit v2.3.0 (ref. ^120^) with default parameters, and then the coding sequence (CDS) alignment was generated by PAL2NAL v14 (ref. ^121^) with the parameter ‘-nomismatch’. Then, a concatenated super matrix of the 13,328 ortholog-based CDS alignment was constructed with different partitions defined corresponding to different gene clusters. The super matrix and partition definition were used for building a maximal likelihood tree using IQ-TREE v1.6.12 (ref. ^122^) (parameters: -m MFP -bb 1000 -alrt 1000 -redo -safe). The SNP list of the 72 accessions was converted into FASTA format using vcf2phylip v2.8 (https://github.com/edgardomortiz/vcf2phylip), and then was taken by IQ-TREE to reconstruct the evolutionary tree (parameters: -m GTR + ASC -bb 1000 -alrt 1000 -redo -safe). The gene presence–absence variation matrix (phylip format) was generated and used for tree building by IQ-TREE (parameters: -st MORPH -m MK + ASC -bb 1000 -alrt 1000 -redo -safe).

To estimate population recombination rates (*ρ* = 4*N*_e_*r*, where *N*_e_ is the effective population size and *r* is the recombination rate of the window), we used FastEPRR v2.0 (ref. ^123^) with 100-kb nonoverlapping window size. The nucleotide diversity of each site was calculated by VCFtools.

To calculate the K_a_, K_s_ and K_a_/K_s_ of each orthologous gene pair (*A. lyrata* as the reference), the amino acid sequences were aligned using MUSCLE, transformed into CDS alignments with PAL2NAL, and then fed into the KaKs_Calculator v2.0 (refs. ^124,125^).

### Gene expression analysis

The RNA-seq dataset from a previous study^126^, including 79 organs and developmental stages of *A. thaliana*, was downloaded from the NCBI SRA database. First, the potential adapter and low-quality sequences were identified and removed by Trimmomatic v0.39 (ref. ^95^), with parameters ‘LEADING:3 TRAILING:3 SLIDINGWINDOW:4:15 MINLEN:36’. Then, HISAT2 v2.1.0 (refs. ^127,128^) was used to align the clean reads against the Col-PEK genome. Gene expression was normalized as TPM, which was calculated by StringTie v2.0.6 (ref. ^129^) with default parameters.

### Reporting summary

Further information on research design is available in the Nature Portfolio Reporting Summary linked to this article.

## Online content

Any methods, additional references, Nature Portfolio reporting summaries, source data, extended data, supplementary information, acknowledgements, peer review information; details of author contributions and competing interests; and statements of data and code availability are available at 10.1038/s41588-024-01715-9.

### Supplementary information

Supplementary Information Supplementary Figs. 1–22.

Reporting Summary

Peer Review File

Supplementary Tables Supplementary Tables 1–11.

### Source data

Source Data Fig. 1 Statistical source data.

Source Data Fig. 2 Statistical source data.

Source Data Fig. 3 Statistical source data.

Source Data Fig. 4 Statistical source data.

Source Data Fig. 5 Statistical source data.

## Figures and Tables

**Extended Data Fig. 5:**
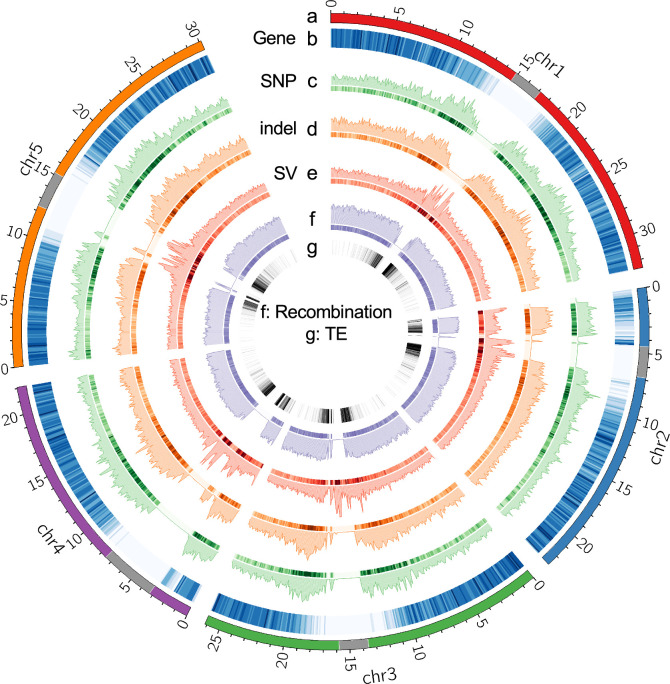
The distribution of genetic variation in the 72 genomes along chromosomes of Col-PEK. Distribution map of genetic variation in the 72 genomes along the chromosomes profiled in 100 kb windows. a: Chromosomes, centromeres are marked by grey; b: Gene density; c: SNP density; d: small indel density; e: SV density (69 accessions); f: historical recombination map (4Ner per kb), centromeres masked; g: TE density.

## Data Availability

The raw Illumina, PacBio HiFi and Oxford Nanopore sequencing data of the 72 accessions can be accessed in EMBL-ENA under the accession number PRJEB62038. The genome assemblies can be accessed in NCBI under the accession number PRJNA1033522. The data generated in this study can be accessed in Edmond (the Open Research Data Repository of the Max Planck Society, 10.17617/3.AEOJBL) (ref. ^130^), including genome assemblies (including Lu-1, Pa-1 and Istisu-1), gene and TE annotations, SNPs and SVs, pan-genome matrix and othrogroups. The RNA-seq dataset used in this study is downloaded from the NCBI SRA database (the accession numbers are included in Supplementary Table 11). The databases used in this study, including OrthoDB brassicales_odb10 (brassicales, 2020-08-05), NCBI NR database and Rfam database v14.8, are all public available. Source data are provided with this paper.

## Code availability

The related code is available at GitHub (https://github.com/qclian/Pan_Ath) and Zenodo (10.5281/zenodo.10567419) (ref. ^131^). All software used in the study are publicly available from the Internet as described in Methods and Reporting Summary.
