# Supplementary material for: A pan-genome of 69 Arabidopsis thaliana accessions reveals a conserved genome structure throughout the global species range
Source: Nat Genet. 2024 Apr 11;56(5):982–91. doi: 10.1038/s41588-024-01715-9 (PMC11096106; doi:10.1038/s41588-024-01715-9)
Supplement: Supplementary file 1 — Supplementary Figs. 1–22. [file 41588_2024_1715_MOESM1_ESM.pdf]

nature genetics

Article

https://doi.org/10.1038/s41588-024-01715-9

A pan-genome of 69 *Arabidopsis thaliana* accessions reveals a conserved genome structure throughout the global species range

In the format provided by the authors and unedited

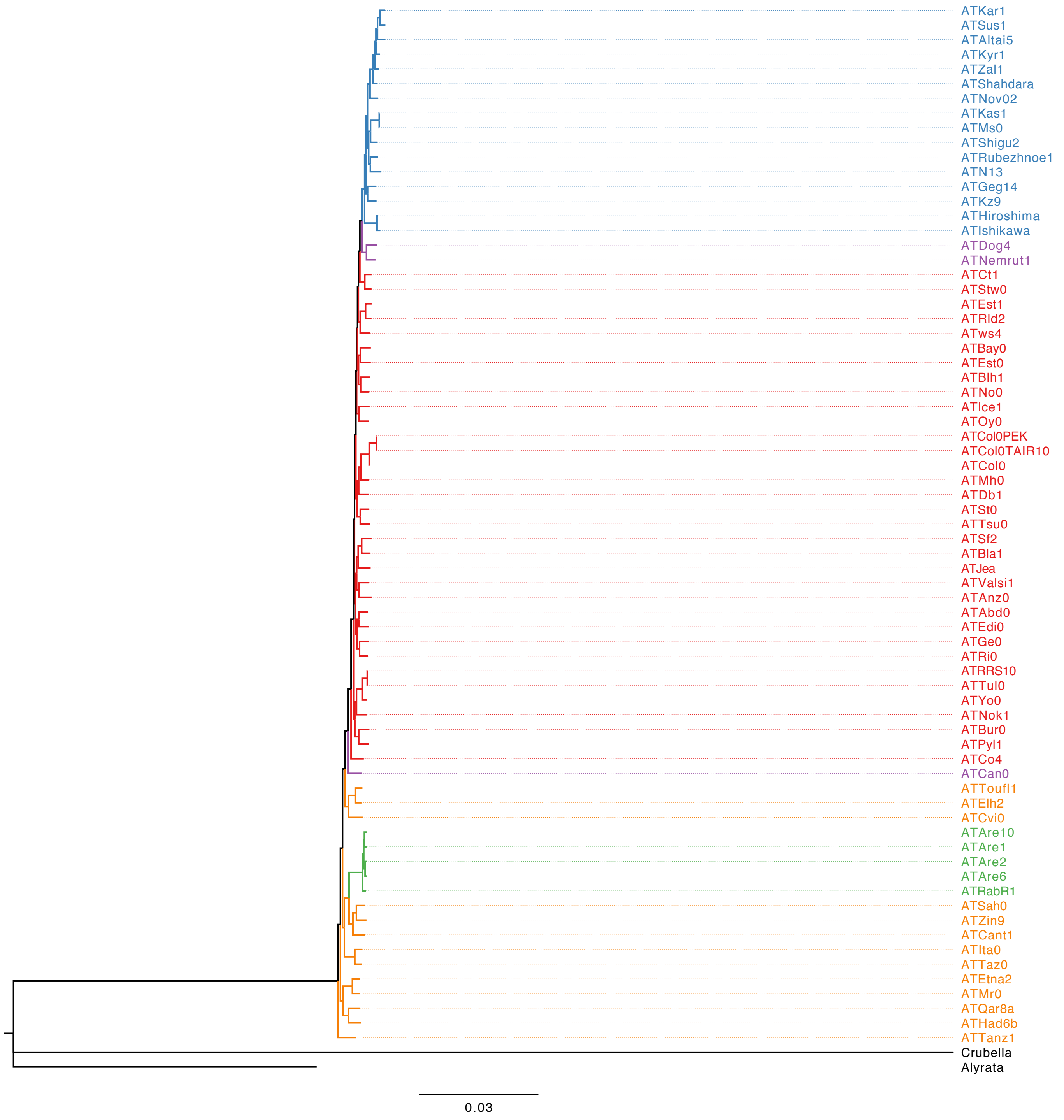

Supplementary Figure 1. Phylogenetic tree based on single-copy orthologues. The tree was rooted by including A. lyrata and C. rubella as outgroups. Tree branches (accessions) are coloured according to the genetic classification. Europe (red), Asia (blue), Madeira (green), Africa (orange) and admixture (purple).

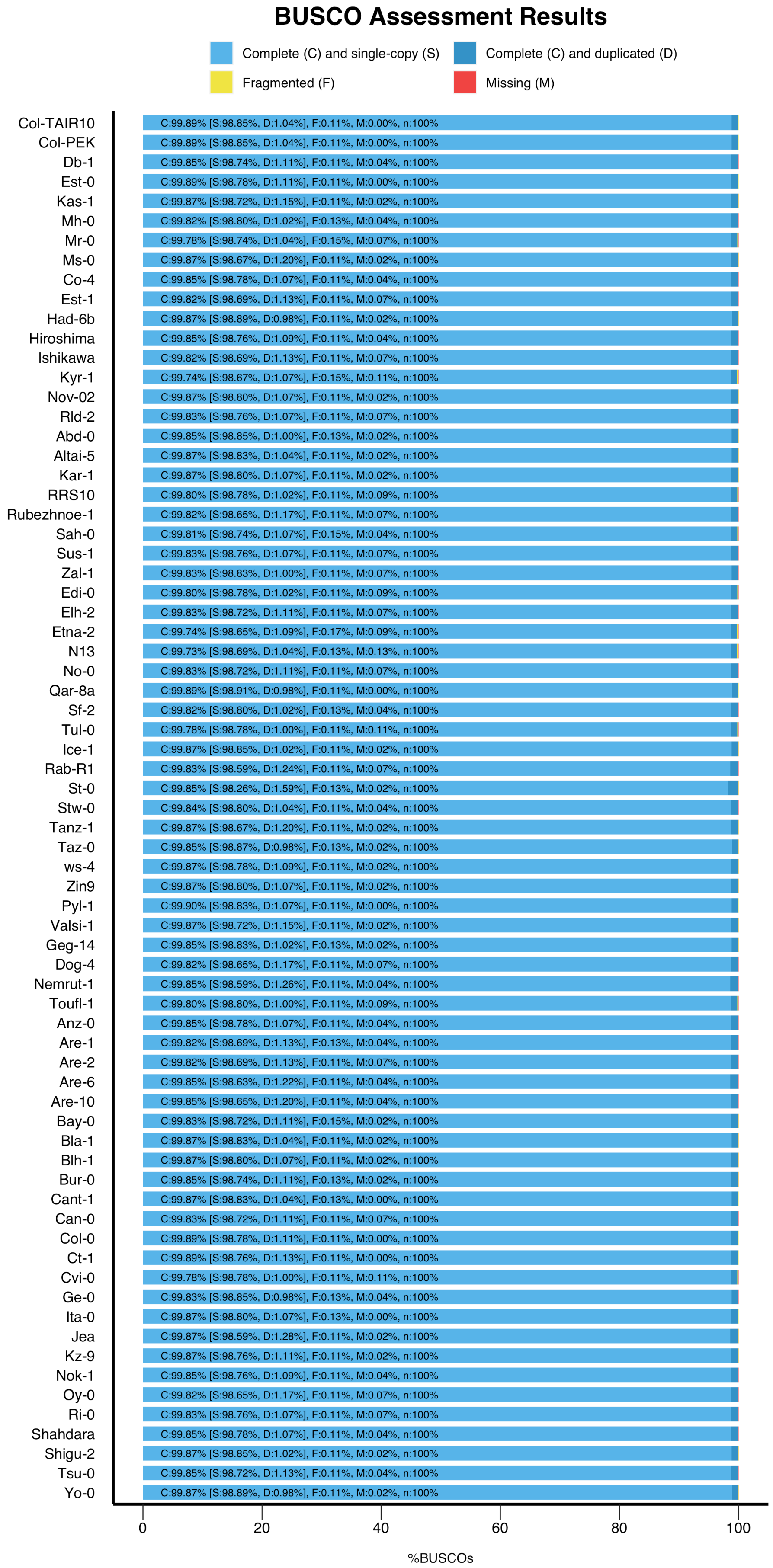

Supplementary Figure 2. BUSCO analysis of the 69 *A. thaliana* genomes. Col-TAIR10 and Col-PEK were also included in the comparison.

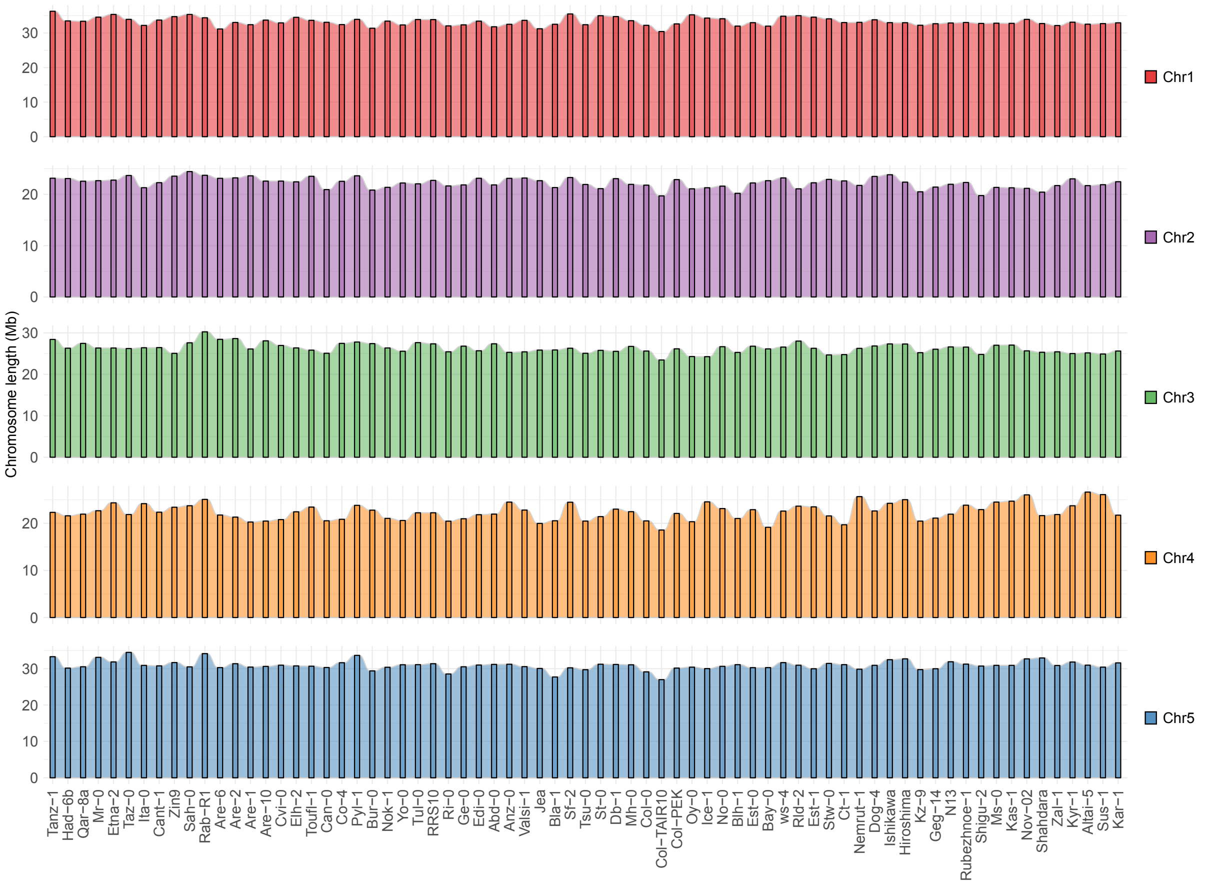

Supplementary Figure 3. The distribution of chromosome lengths of the 69 *A. thaliana* genomes.

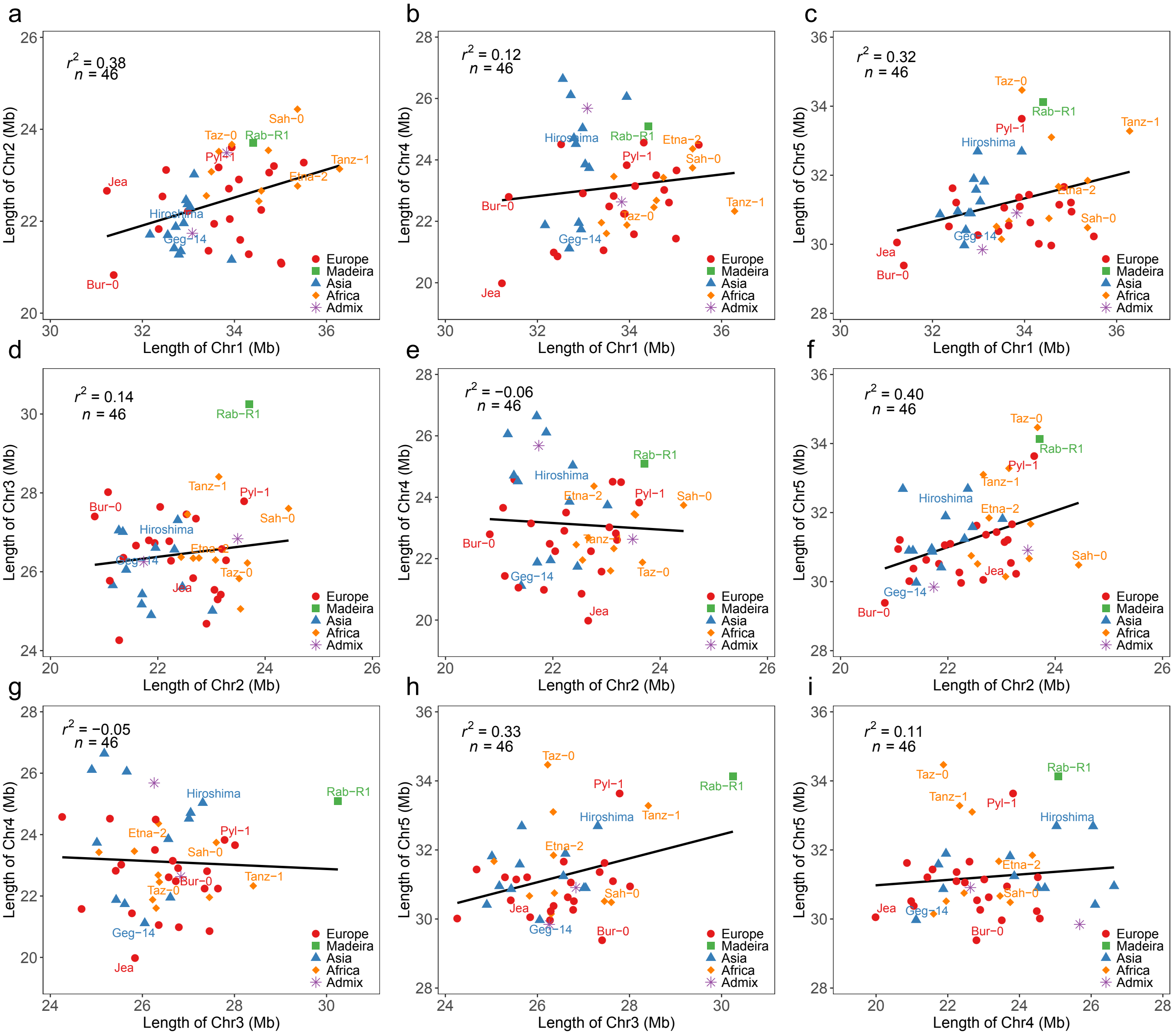

Supplementary Figure 4. Correlation analysis of chromosome lengths of the 46 *A. thaliana* genomes.

Comparison of assembly length between chromosomes in the 46 accessions with the most complete genome assembly. Pearson's correlation analysis was performed, and the coefficient is shown in each panel. Accessions are coloured according to their genetic classification.

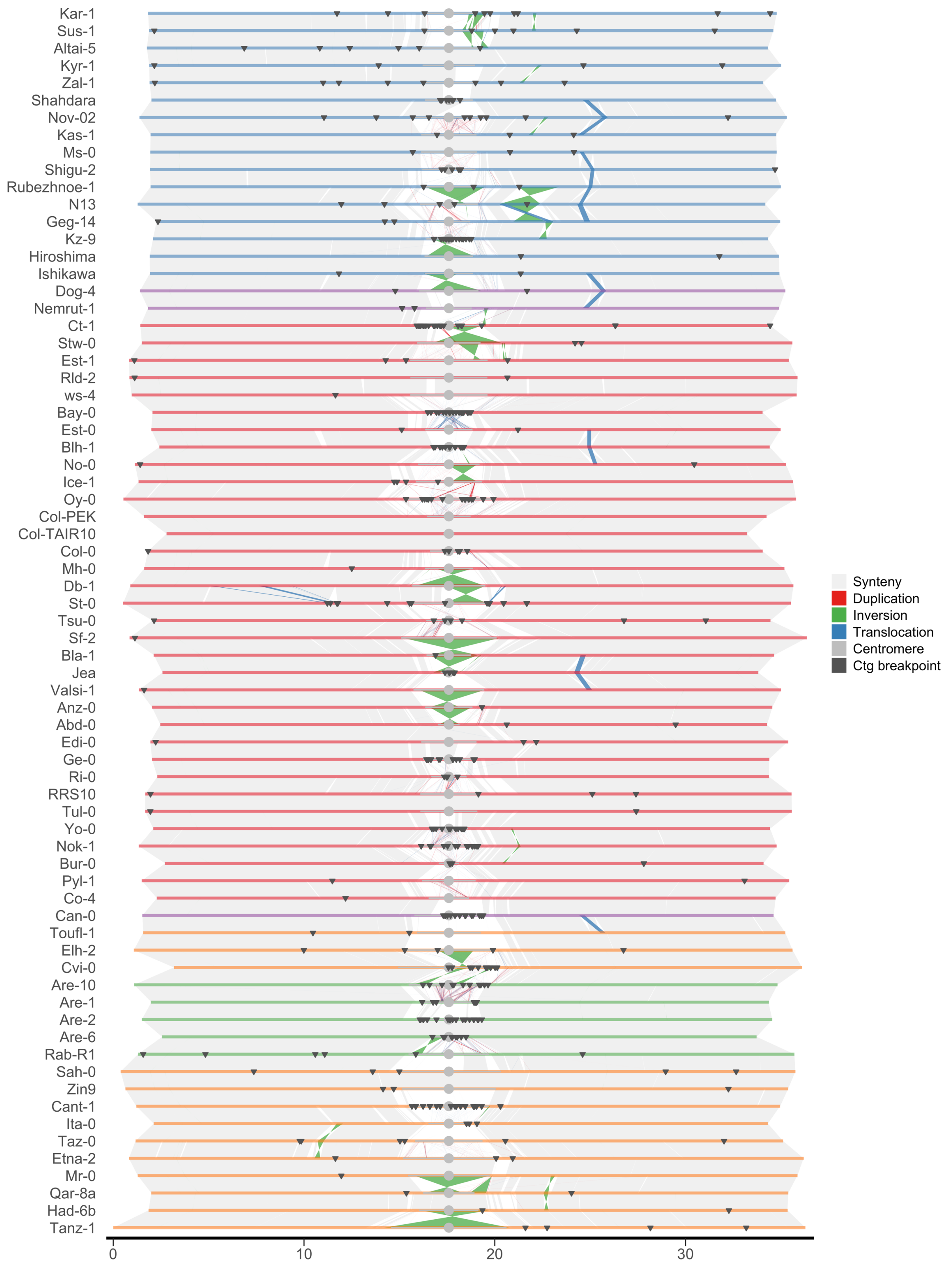

Supplementary Figure 5. Whole-chromosome alignment of chromosome 1 from the 69 *A. thaliana* genomes.

Chromosomes are represented by segments, which are colored according to their genetic classification. The grey segments represent the centromeric regions, and the middle points are indicated by the grey circles. The syntenic regions between chromosomes from 69 genomes are colored by light grey. The structural rearrangements, including duplications, inversions and translocations, are colored red, green and blue, respectively. The contig breakpoints are marked by black triangles.

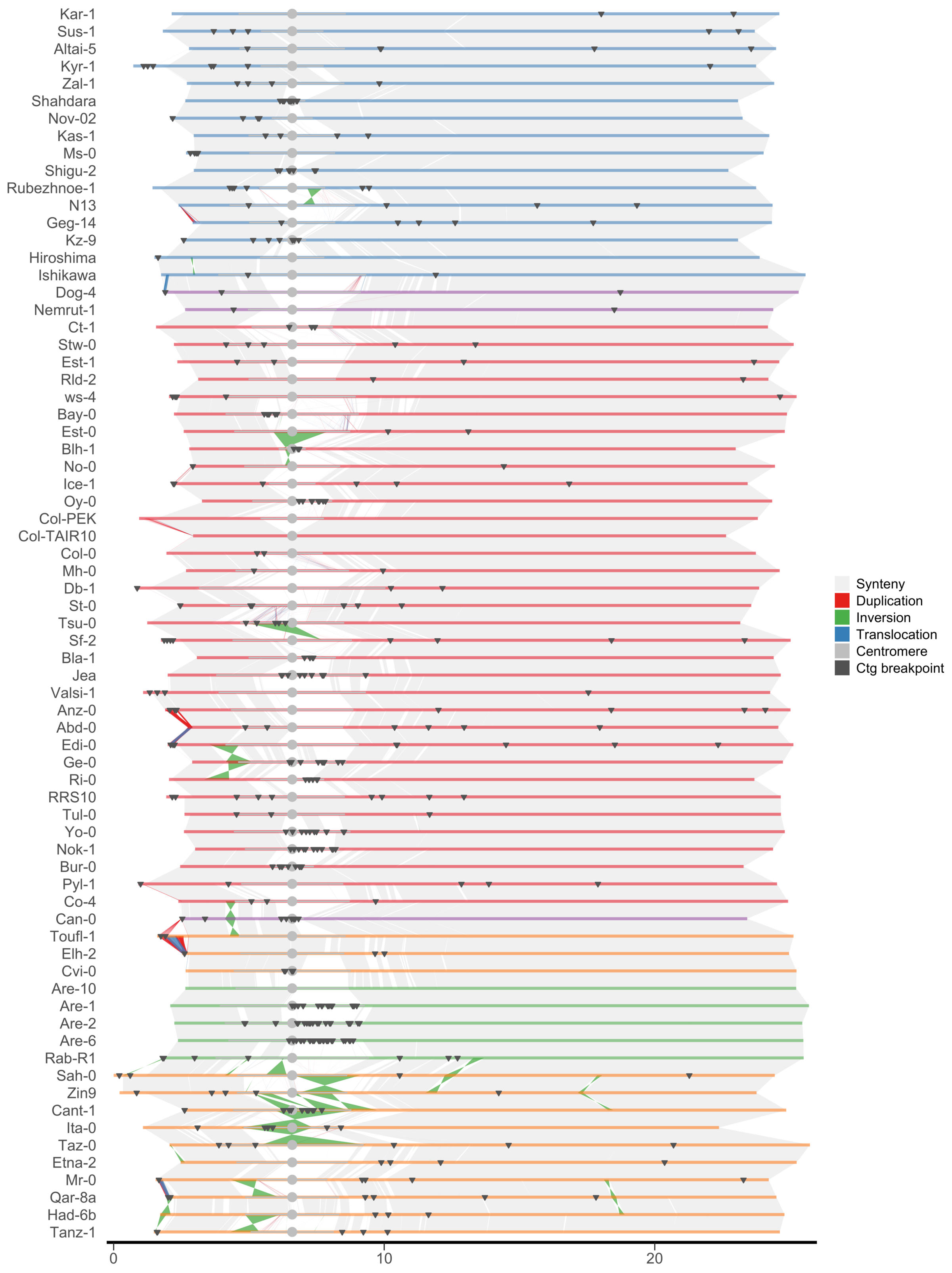

Supplementary Figure 6. Whole-chromosome alignment of chromosome 2 from the 69 *A. thaliana* genomes.

Chromosomes are represented by segments, which are colored according to their genetic classification. The grey segments represent the centromeric regions, and the middle points are indicated by the grey circles. The syntenic regions between chromosomes from 69 genomes are colored by light grey. The structural rearrangements, including duplications, inversions and translocations, are colored red, green and blue, respectively. The contig breakpoints are marked by black triangles.

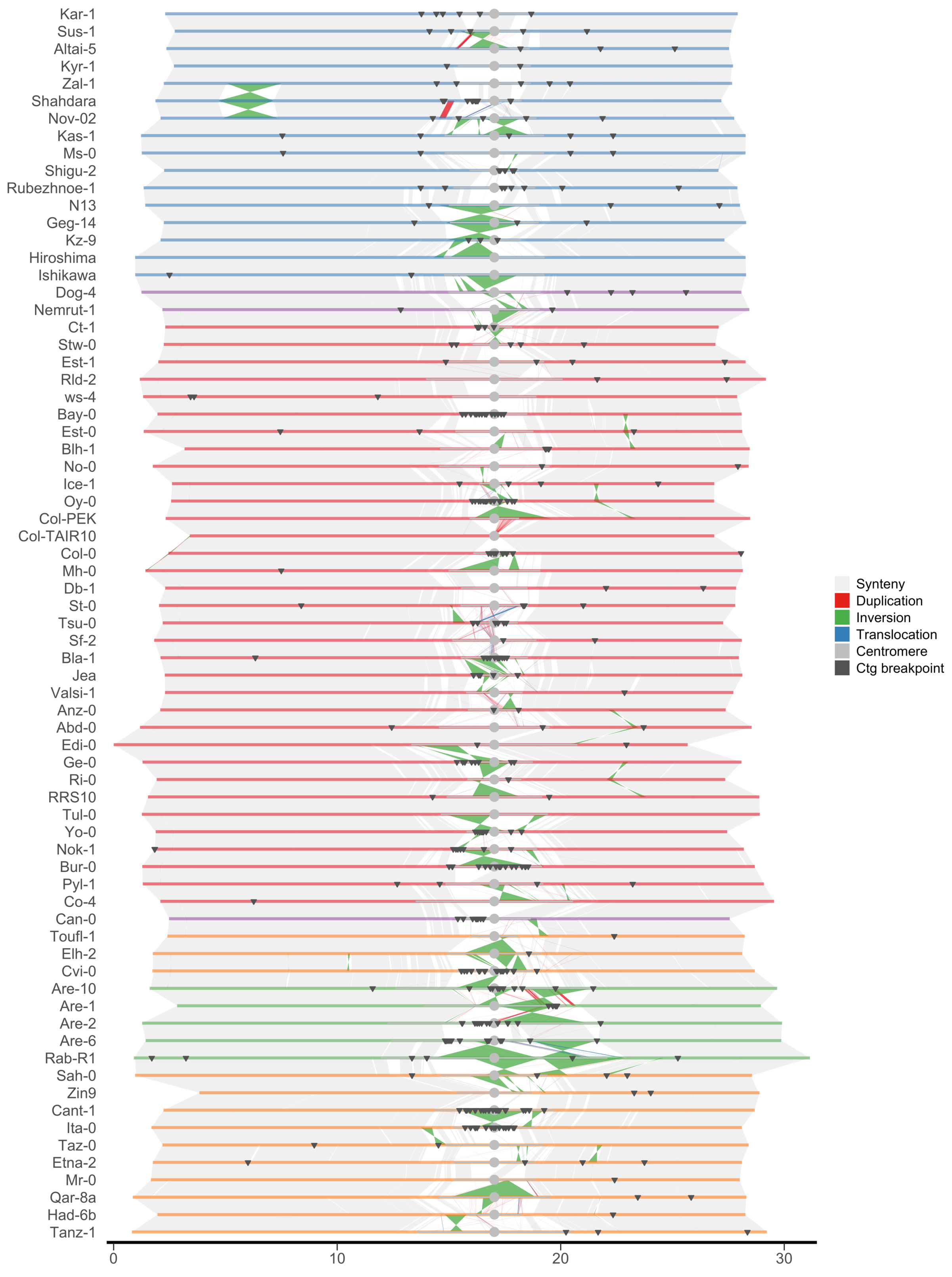

Supplementary Figure 7. Whole-chromosome alignment of chromosome 3 from the 69 *A. thaliana* genomes.

Chromosomes are represented by segments, which are colored according to their genetic classification. The grey segments represent the centromeric regions, and the middle point are indicated by the grey circles. The syntenic regions between chromosomes from 72 genomes are colored by light grey. The structural rearrangements, including duplications, inversions and translocations, are colored red, green and blue, respectively. The contig breakpoints are marked by black triangles.

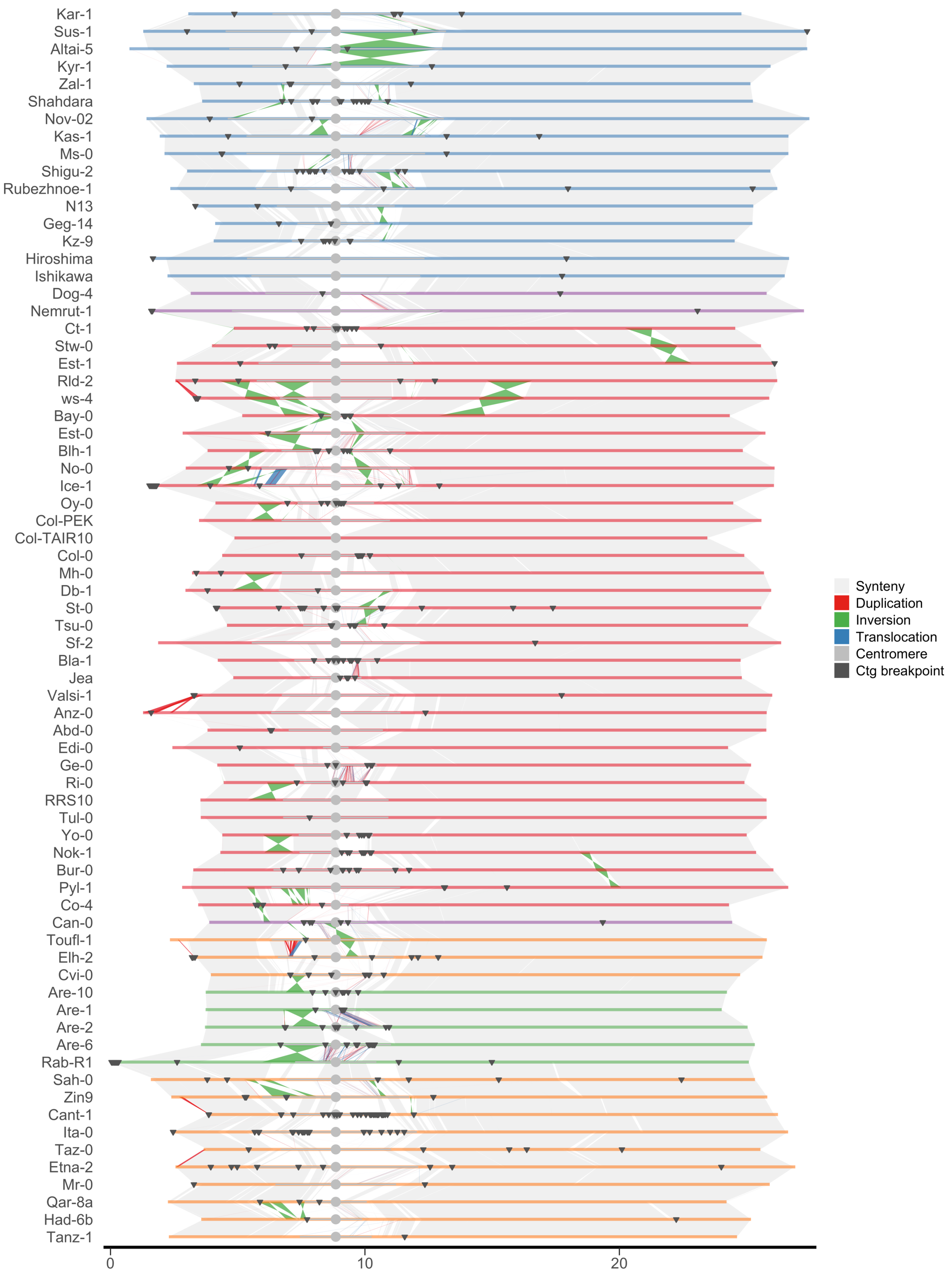

Supplementary Figure 8. Whole-chromosome alignment of chromosome 4 from the 69 *A. thaliana* genomes.

Chromosomes are represented by segments, which are colored according to their genetic classification. The grey segments represent the centromeric regions, and the middle points are indicated by the grey circles. The syntenic regions between chromosomes from 69 genomes are colored by light grey. The structural rearrangements, including duplications, inversions and translocations, are colored red, green and blue, respectively. The contig breakpoints are marked by black triangles.

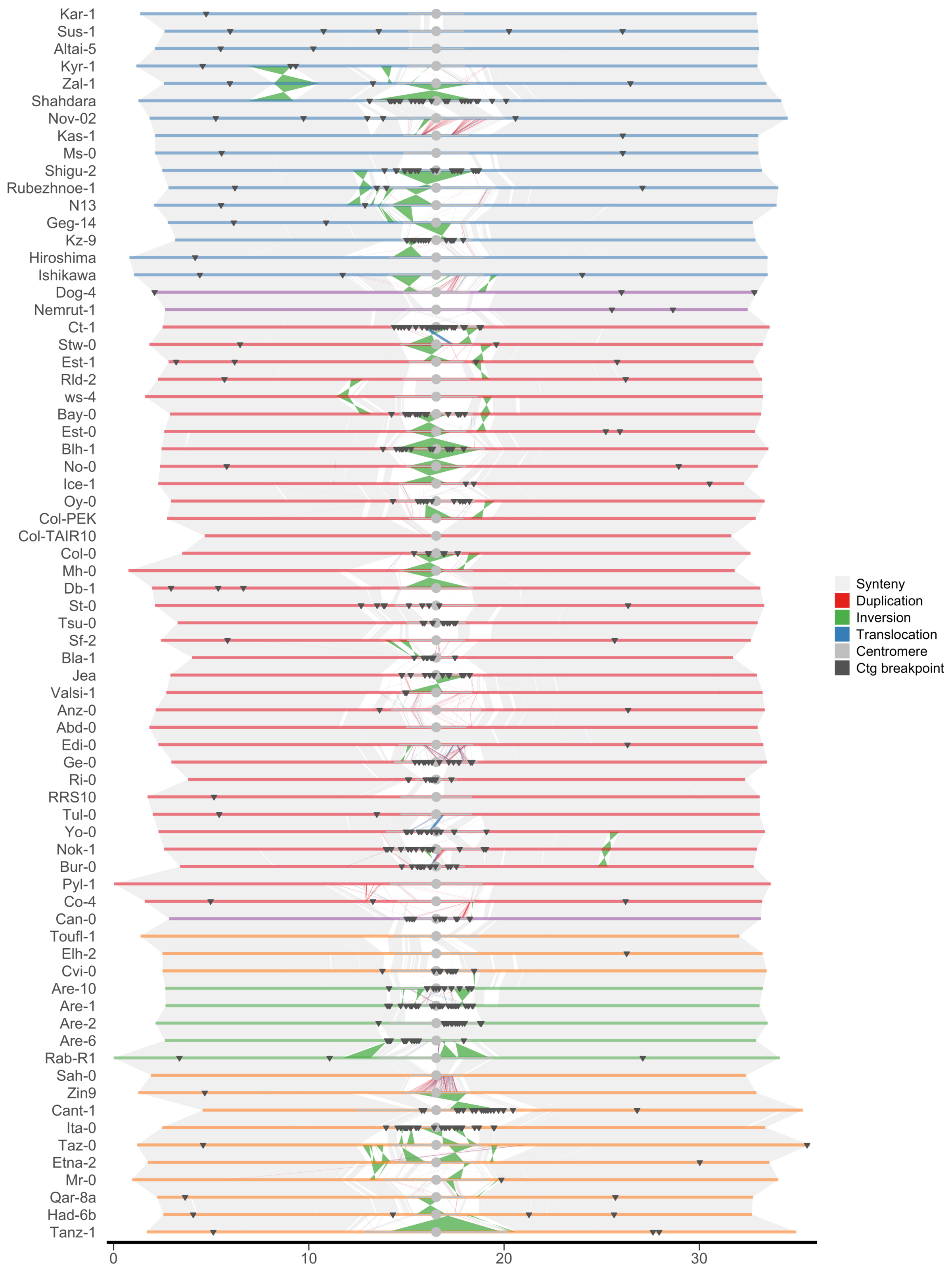

Supplementary Figure 9. Whole-chromosome alignment of chromosome 5 from the 69 *A. thaliana* genomes.

Chromosomes are represented by segments, which are colored according to their genetic classification. The grey segments represent the centromeric regions, and the middle points are indicated by the grey circles. The syntenic regions between chromosomes from 69 genomes are colored by light grey. The structural rearrangements, including duplications, inversions and translocations, are colored red, green and blue, respectively. The contig breakpoints are marked by black triangles.

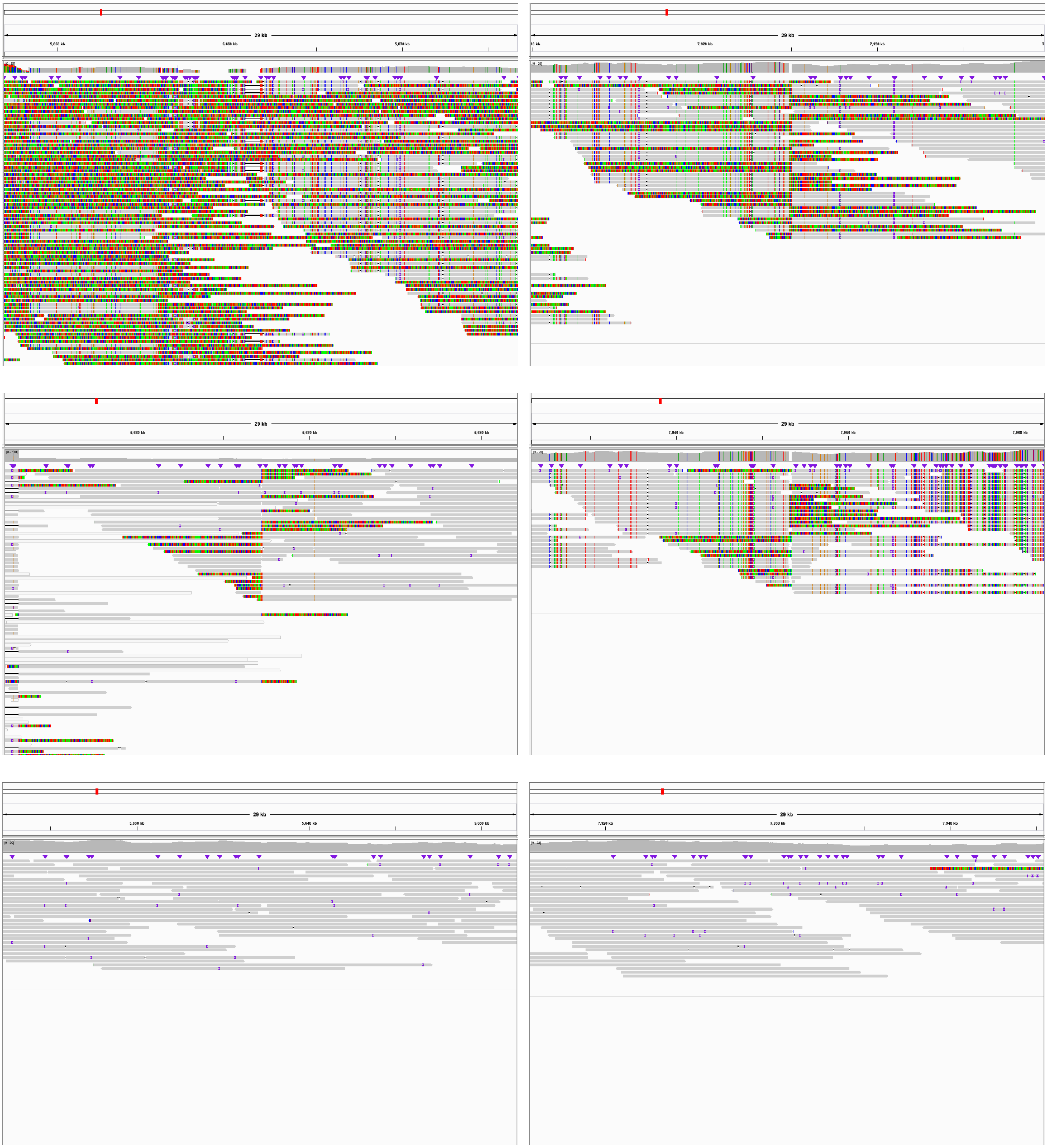

Supplementary Figure 10. Alignment of long-reads in the breakpoint regions of the inversion in Zal-1.

The top, middle and bottom panels show the alignments of long-reads from Zal-1 against Col-PEK, the closest accession Kyr-1, and the Zal-1 assemblies, with a window of 30 kb covering the left and right breakpoints of the detected inversion, respectively.

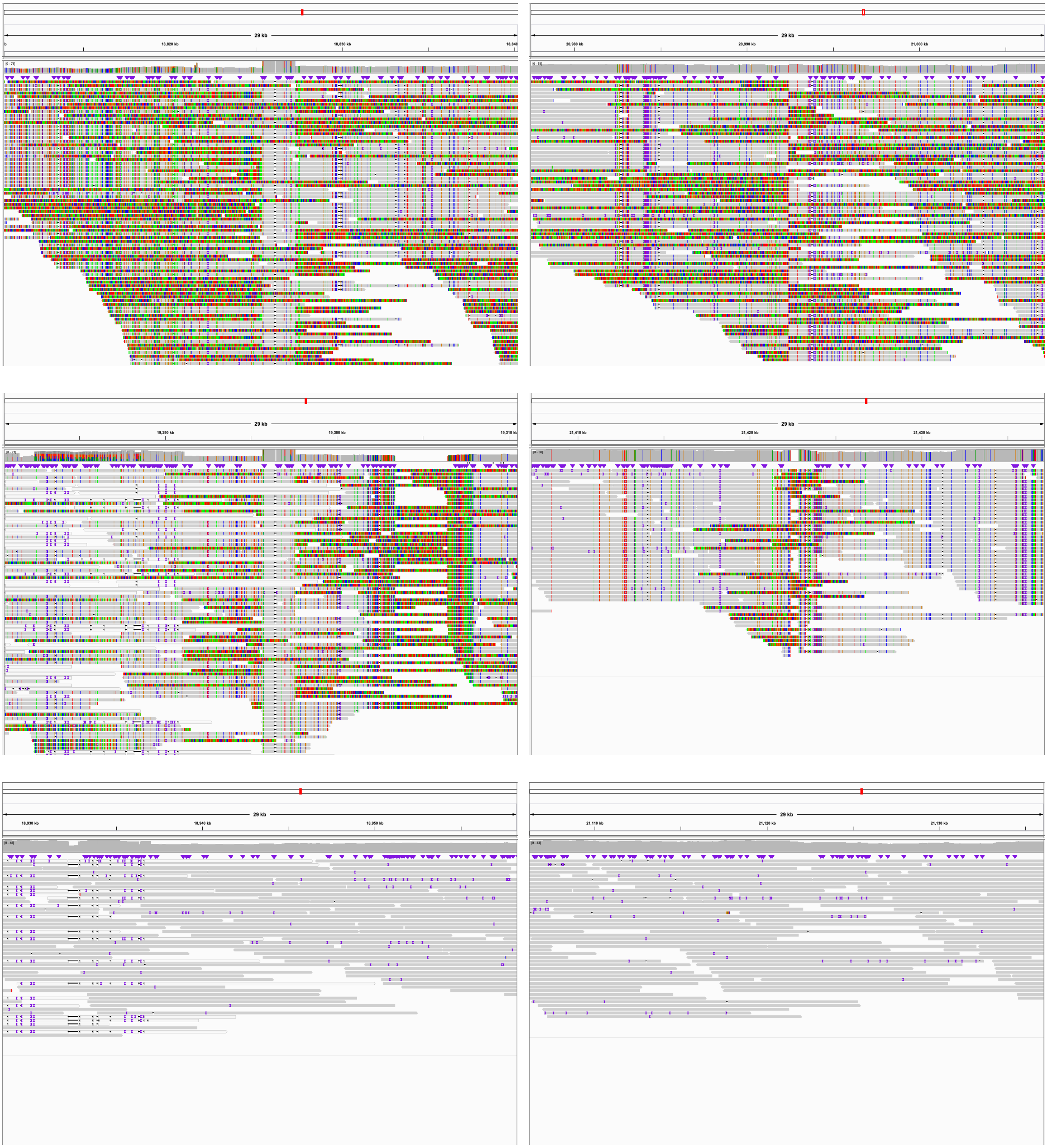

Supplementary Figure 11. Alignment of long-reads in the breakpoint regions of the inversion in N13.

The top, middle and bottom panels show the alignments of long-reads from N13 against Col-PEK, the closest accession Rubezhnoe-1, and the N13 assemblies, with a window of 30 kb covering the left and right breakpoints of the detected inversion, respectively.

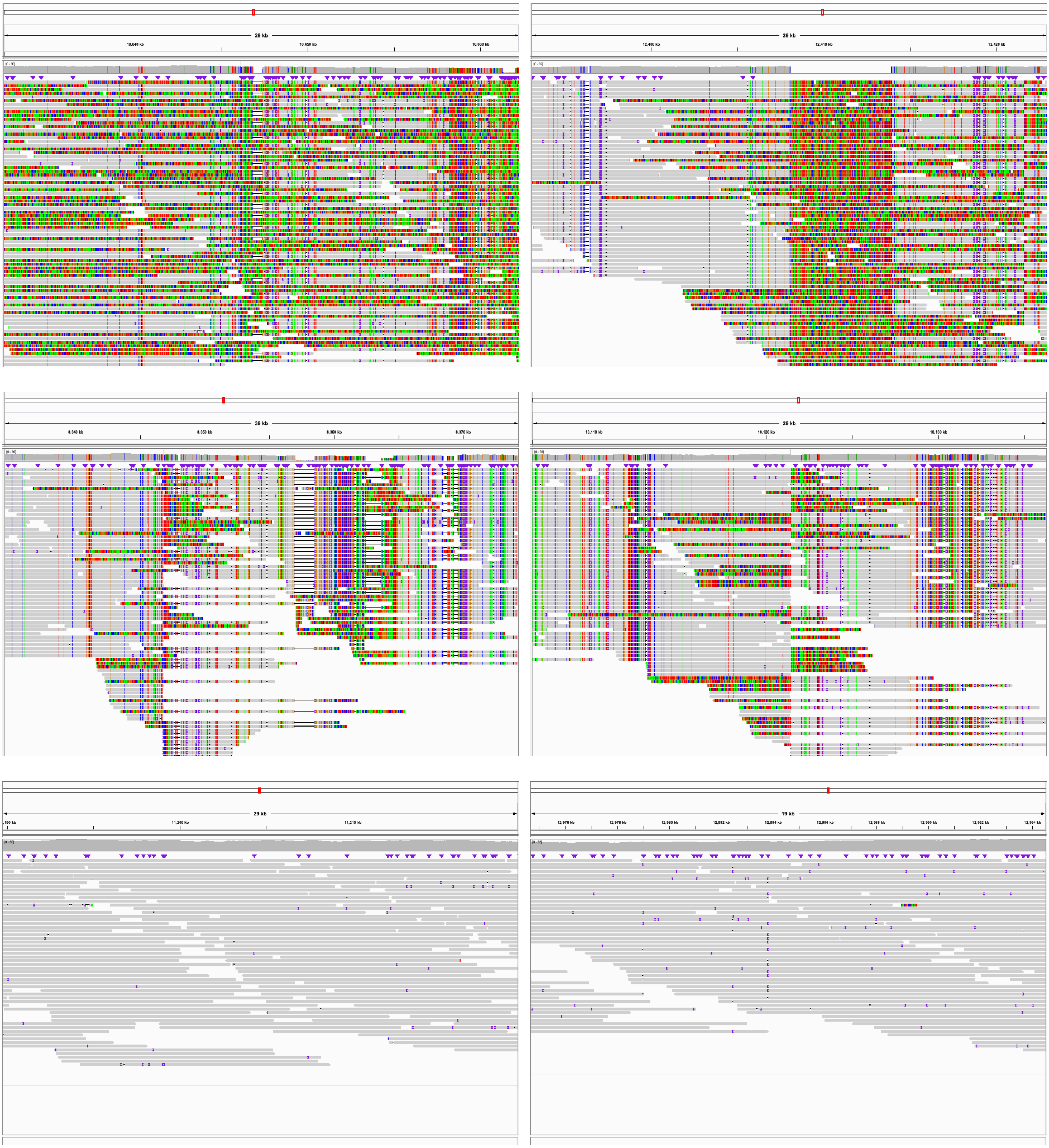

Supplementary Figure 12. Alignment of long-reads in the breakpoint regions of the inversion in ws-4.

The top, middle and bottom panels show the alignments of long-reads from ws-4 against Col-PEK, the closest accession Ct-1, and the ws-4 assemblies, with a window of 30 kb covering the left and right breakpoints of the detected inversion, respectively.

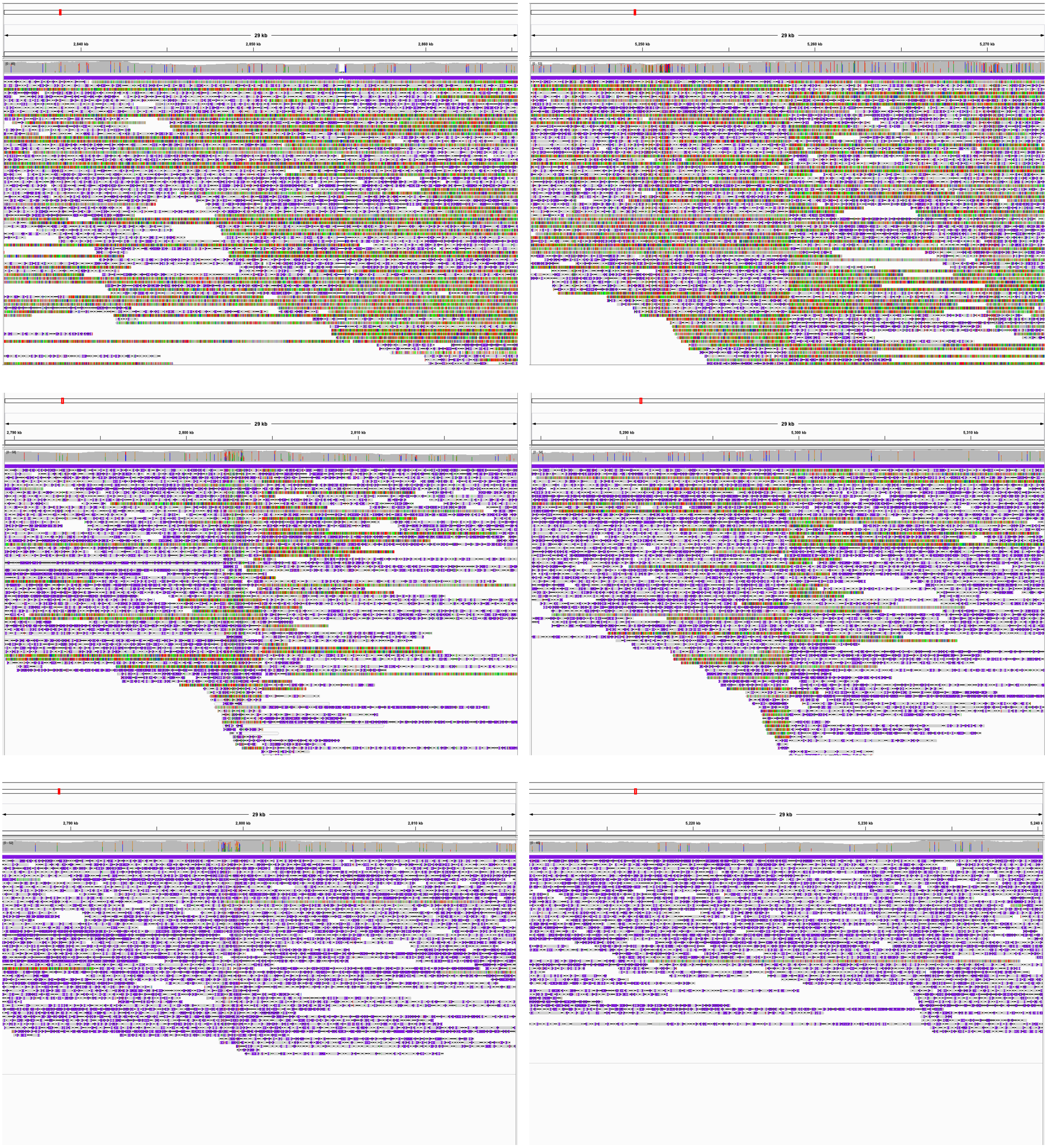

Supplementary Figure 13. Alignment of long-reads in the breakpoint regions of the inversion in Shahdara.

The top, middle and bottom panels show the alignment of long-reads from Shahdara against Col-PEK, the closest accession Kyr-1, and the Shahdara assemblies, with a window of 30 kb covering the left and right breakpoints of the detected inversion, respectively.

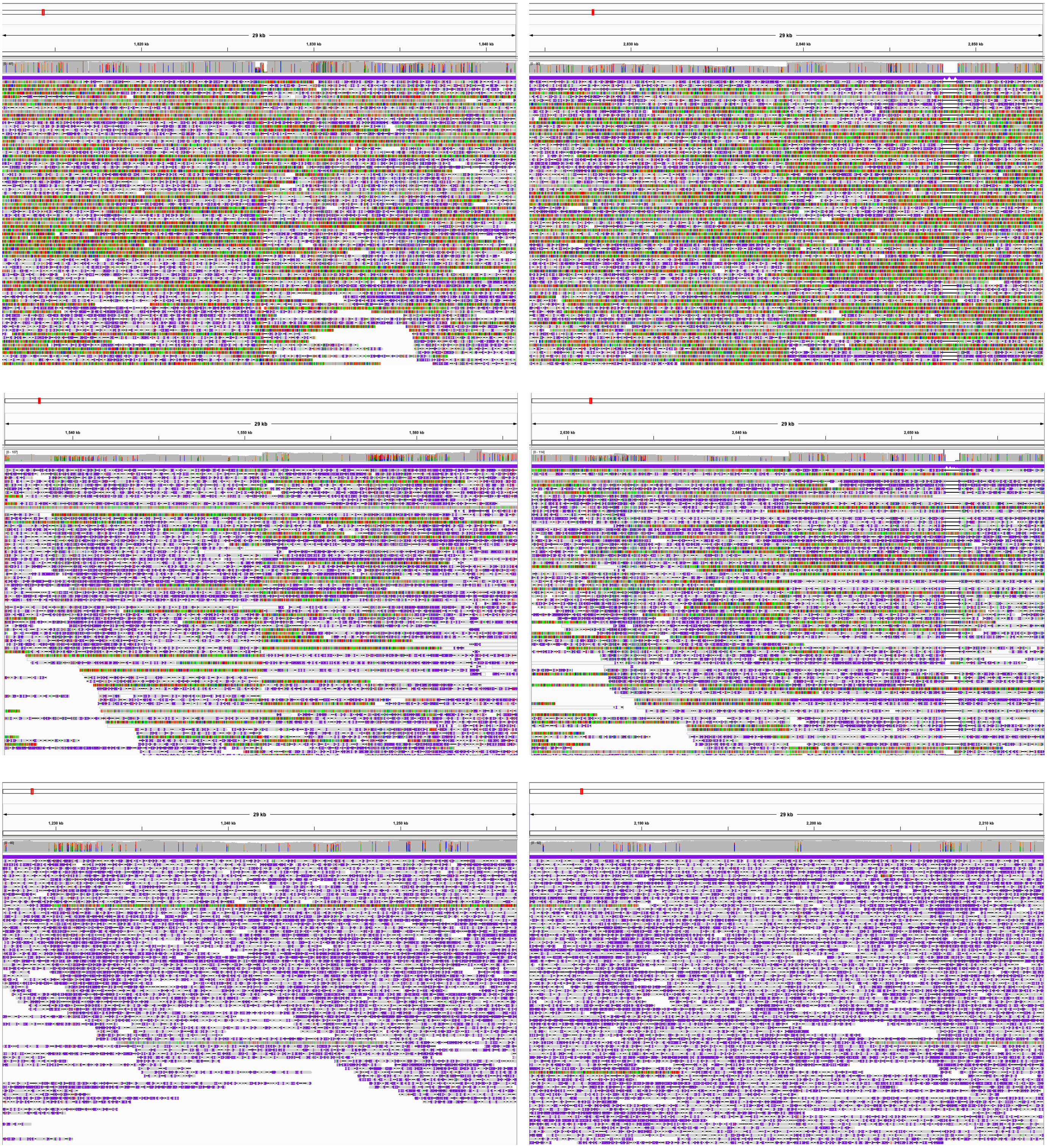

Supplementary Figure 14. Alignment of long-reads in the breakpoint regions of the inversion in Ge-0.

The top, middle and bottom panels show the alignment of long-reads from Ge-0 against Col-PEK, the closest accession Edi-0, and the Ge-0 assemblies, with a window of 30 kb covering the left and right breakpoints of the detected inversion, respectively.

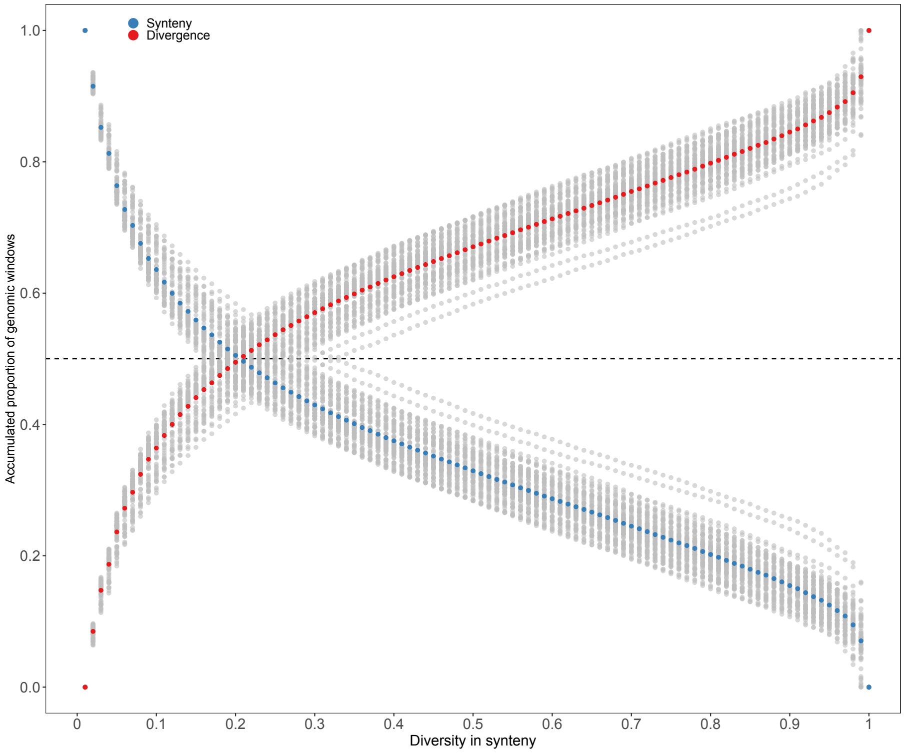

Supplementary Figure 15. Frequency of diversity in synteny in the population of 69 *A. thaliana* accessions.

The diversity in synteny was calculated for each of the 69 genomes using 10 kb non-overlapping genomic windows. For each genome, accumulated proportion of genomic windows with diversity in synteny, ranging from 0 to 1 with bin size of 0.01, was calculated. The mean of each bin of diversity in synteny was presented by red and blue points.

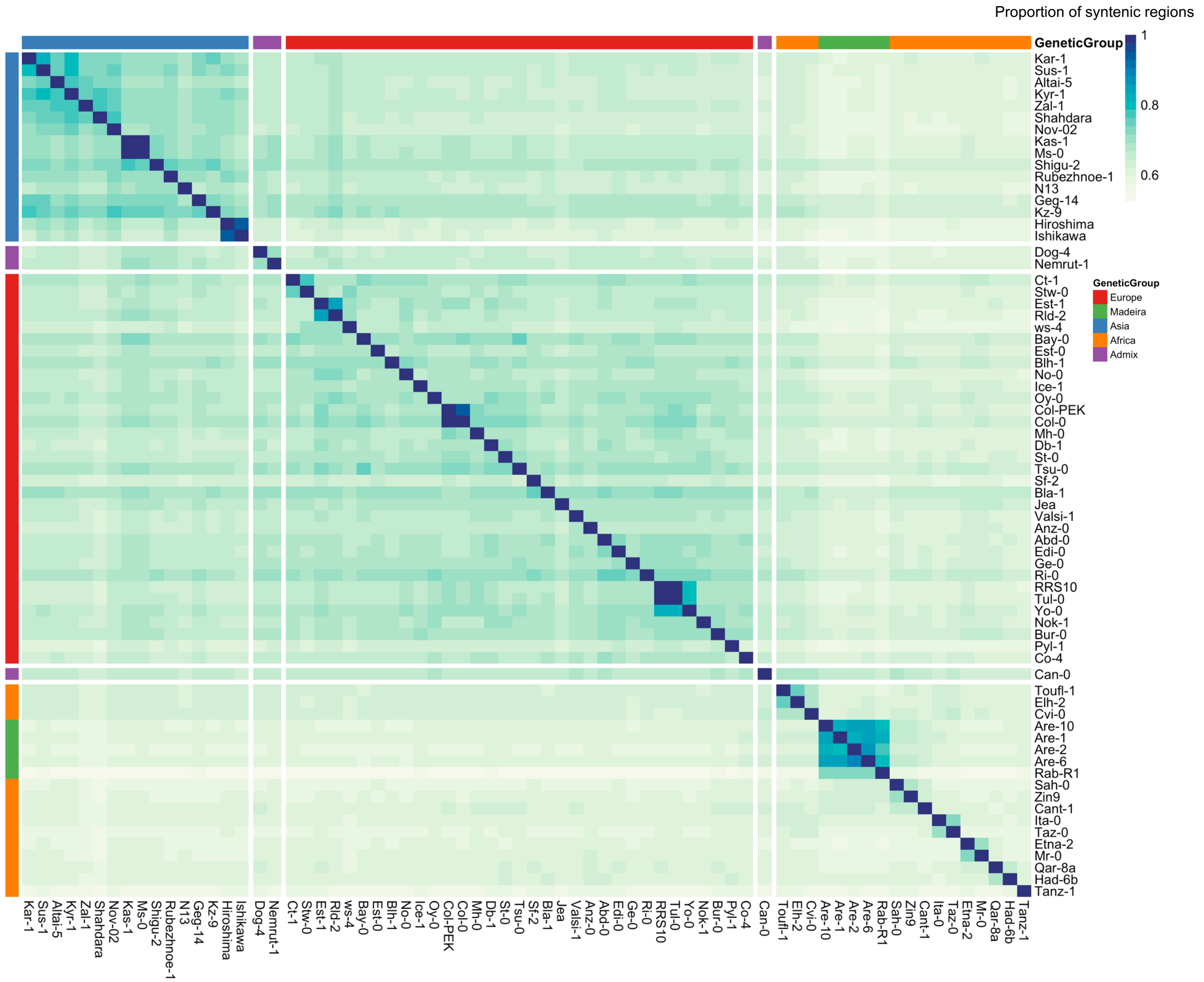

Supplementary Figure 16. Pair-wise synteny relationship measurement along whole chromosomes.

For each pair of genomes, synteny was measured as proportion of syntenic regions. Accessions are coloured according to the genetic classification.

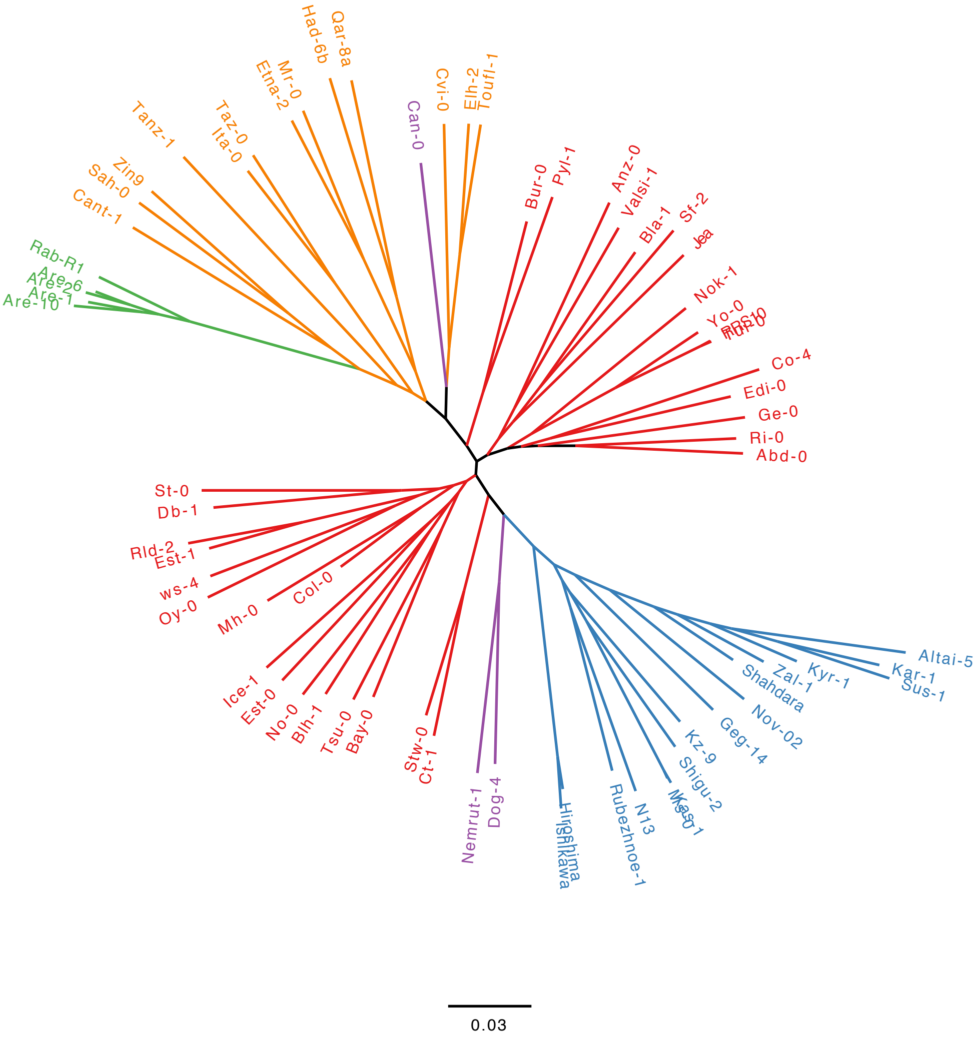

Supplementary Figure 17. Phylogenetic tree based on SVs in the 69 *A. thaliana* genomes.

Tree branches (accessions) are coloured according to their genetic classification. Europe (red), Asia (blue), Madeira (green), Africa (orange) and admixture (purple).

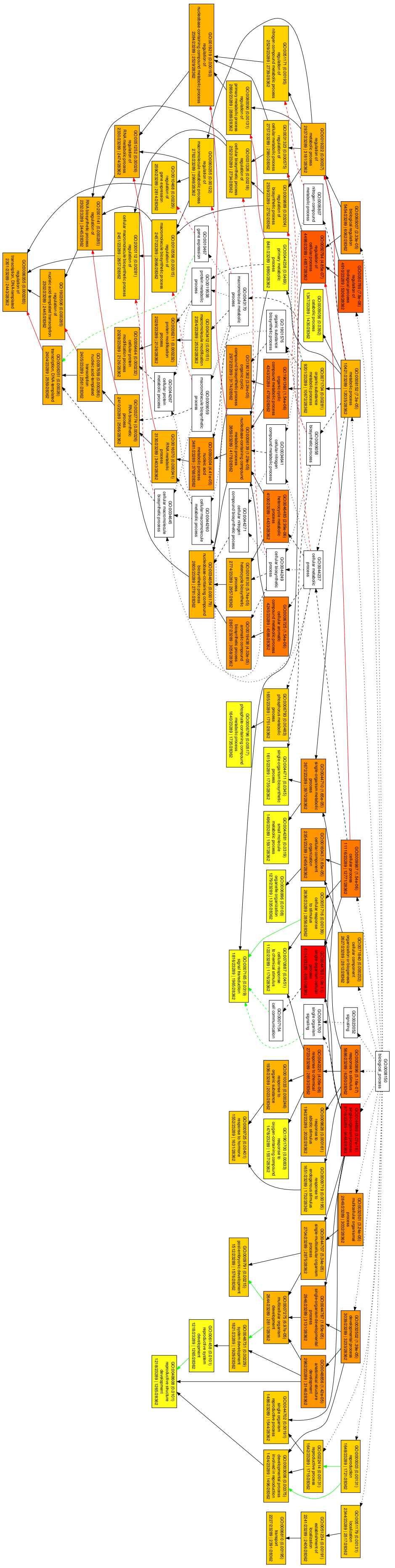

Supplementary Figure 18. GO enrichment analysis of core gene families in the 69 *A. thaliana* genomes.

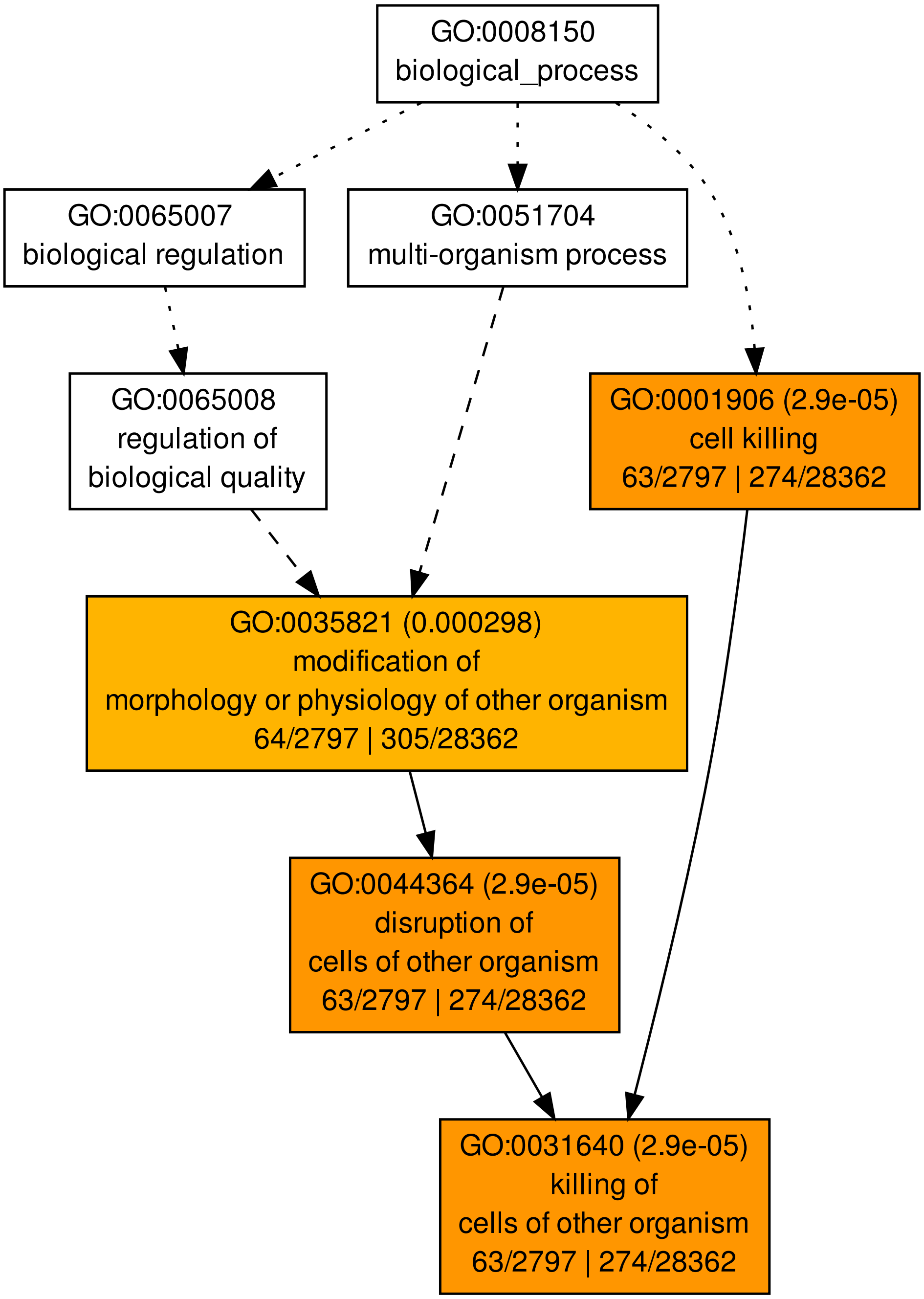

Supplementary Figure 19. GO enrichment analysis of softcore, dispensable and private gene families in the 69 *A. thaliana* genomes.

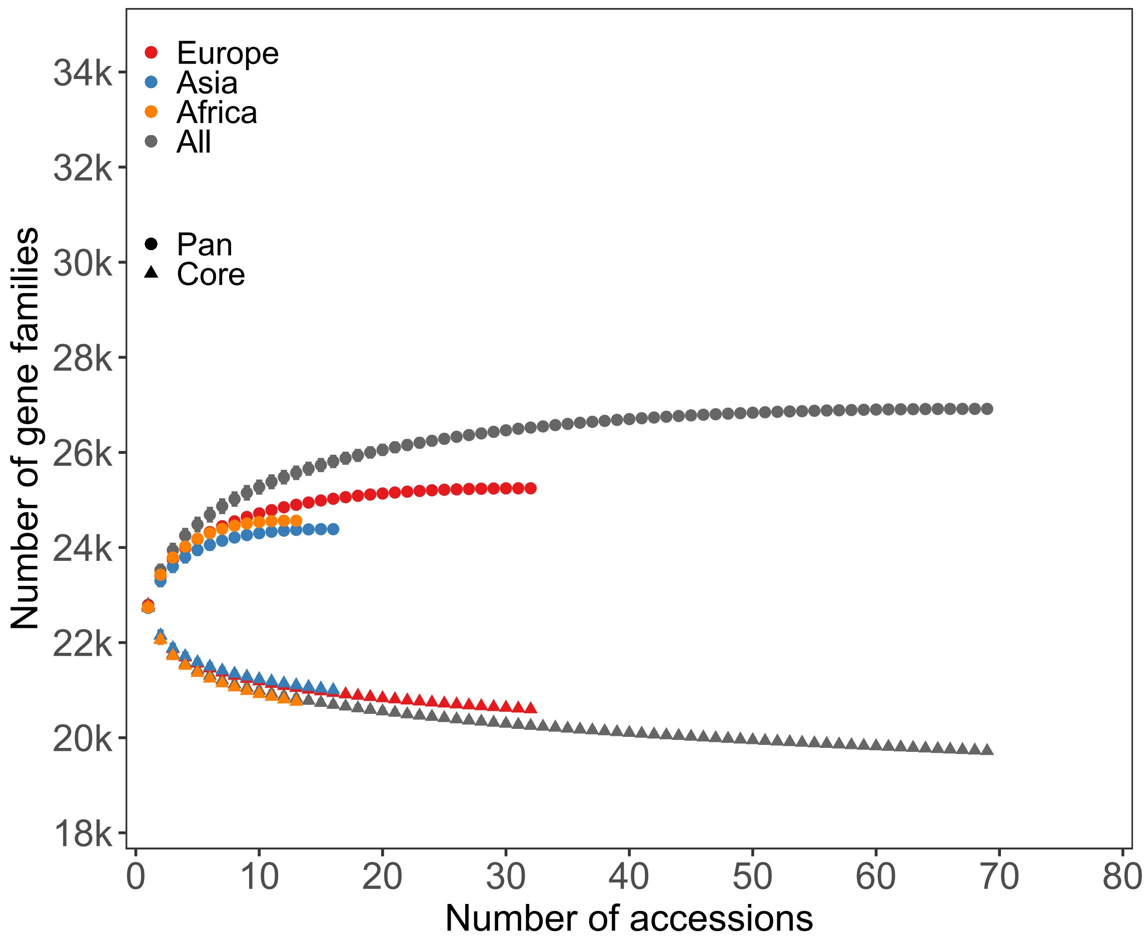

Supplementary Figure 20. Pan-genome estimations for gene space without private genes.

The increase of the pan-genome size and the decrease of core-genome size in the whole population (grey), Europe (red), Asia (blue) and Africa (orange) groups. Accessions were sampled as 2,000 random combinations for each set of accessions of different size ranging from 2 to 67 accessions per set. The mean number of gene families is shown with the standard deviation.

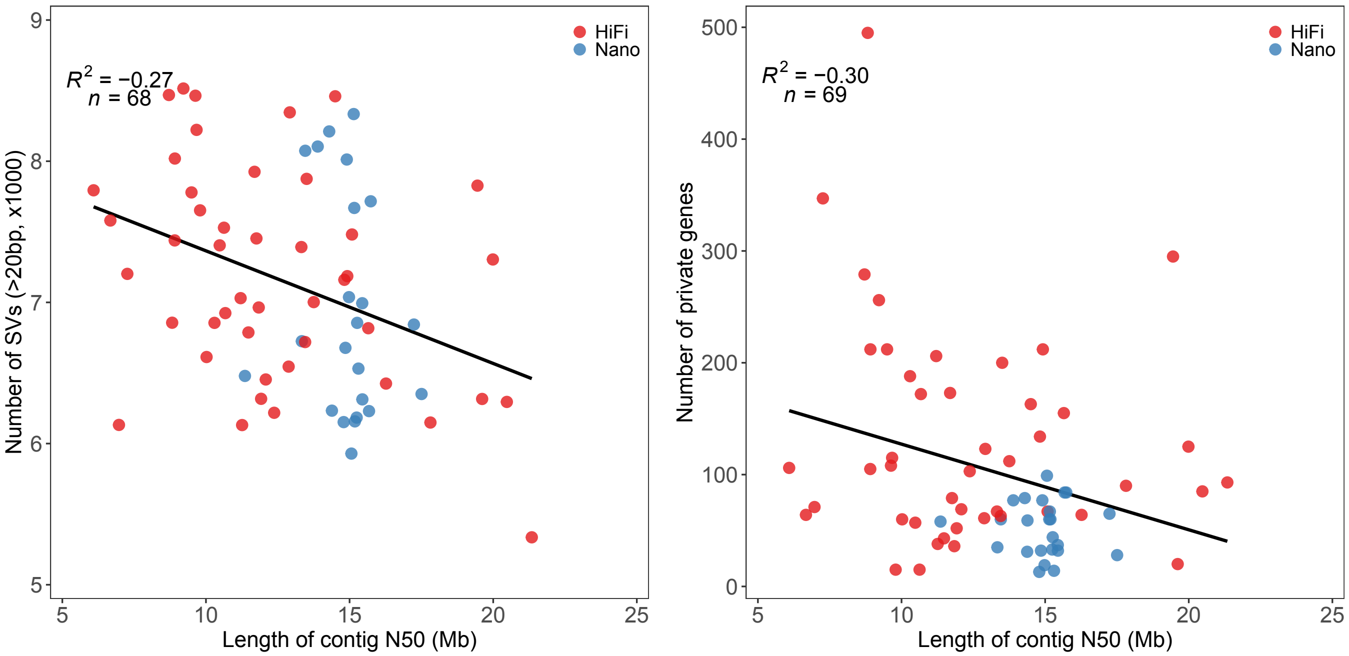

Supplementary Figure 21. The correlation analysis between contig N50 and the number of SVs and private genes.

The accession Col-0 was removed for the comparison between contig N50 and the number of SVs. Accessions sequenced by PacBio HiFi and Oxford Nanopore were coloured in red and blue, respectively. The Pearson's correlation coefficient and the sample size were shown accordingly.

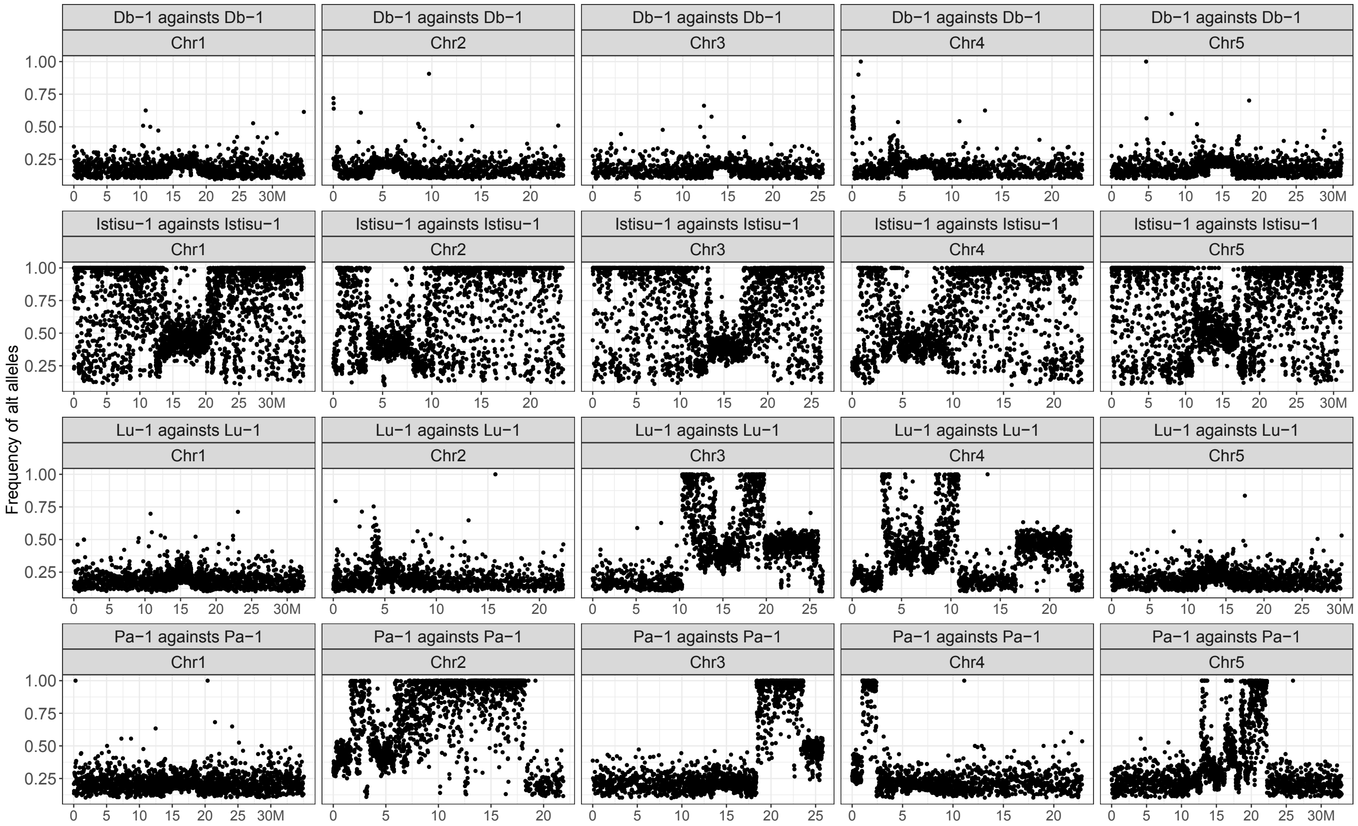

Supplementary Figure 22. The allele frequency along chromosomes in Db-1, Lu-1, Pa-1 and Istisu-1.

The alternative allele frequency was calculated for each 10 kb window.
