# Supplementary material for: A pan-genome of 69 Arabidopsis thaliana accessions reveals a conserved genome structure throughout the global species range
Source: Nat Genet. 2024 Apr 11;56(5):982–91. doi: 10.1038/s41588-024-01715-9 (PMC11096106; doi:10.1038/s41588-024-01715-9)
Supplement: Supplementary file 3 — Peer Review File [file 41588_2024_1715_MOESM3_ESM.pdf]

naturereseach

Peer Review Information

Journal: Nature Genetics
Manuscript Title: A pan-genome of 69 Arabidopsis thaliana accessions reveals a conserved genome structure throughout the global species range
Corresponding author name(s): Professor Korbinian Schneeberger, Professor Raphael Mercier

Reviewer Comments & Decisions:

Decision Letter, initial version:

17th Jul 2023

Dear Professor Mercier,

Your Article, "A pan-genome of 72 Arabidopsis thaliana accessions reveals a conserved genome structure throughout the global species range" has now been seen by 3 referees. You will see from their comments below that while they find your work of interest, some important points are raised. We are interested in the possibility of publishing your study in Nature Genetics, but would like to consider your response to these concerns in the form of a revised manuscript before we make a final decision on publication.

To guide the scope of the revisions, the editors discuss the referee reports in detail within the team with a view to identifying key priorities that should be addressed in revision. In this case, we think all three referees have provided constructive reviews aimed at strengthening the analyses and improving the presentation. We particularly ask that you perform additional analyses as suggested, deposit the genome assemblies and additional files to public repositories, and address all referee comments as thoroughly as possible with appropriate revisions. We hope that you will find the prioritized set of referee points to be useful when revising your study.

We therefore invite you to revise your manuscript taking into account all reviewer and editor comments. Please highlight all changes in the manuscript text file. At this stage we will need you to upload a copy of the manuscript in MS Word .docx or similar editable format.

We are committed to providing a fair and constructive peer-review process. Do not hesitate to contact us if there are specific requests from the reviewers that you believe are technically impossible or unlikely to yield a meaningful outcome.

When revising your manuscript:

*1) Include a “Response to referees” document detailing, point-by-point, how you addressed each referee comment. If no action was taken to address a point, you must provide a compelling argument. This response will be sent back to the referees along with the revised manuscript.

*2) If you have not done so already please begin to revise your manuscript so that it conforms to our Article format instructions, available
here.
Refer also to any guidelines provided in this letter.

*3) Include a revised version of any required Reporting Summary:
https://www.nature.com/documents/nr-reporting-summary.pdf
It will be available to referees (and, potentially, statisticians) to aid in their evaluation if the manuscript goes back for peer review.
A revised checklist is essential for re-review of the paper.

Please be aware of our guidelines on digital image standards.

Please use the link below to submit your revised manuscript and related files:

[redacted]

Note: This URL links to your confidential home page and associated information about manuscripts you may have submitted, or that you are reviewing for us. If you wish to forward this email to co-authors, please delete the link to your homepage.

We hope to receive your revised manuscript within 3 to 6 months. If you cannot send it within this time, please let us know.

Please do not hesitate to contact me if you have any questions or would like to discuss these revisions further.

Nature Genetics is committed to improving transparency in authorship. As part of our efforts in this direction, we are now requesting that all authors identified as ‘corresponding author’ on published papers create and link their Open Researcher and Contributor Identifier (ORCID) with their account on the Manuscript Tracking System (MTS), prior to acceptance. ORCID helps the scientific community achieve unambiguous attribution of all scholarly contributions. You can create and link your ORCID from the home page of the MTS by clicking on ‘Modify my Springer Nature account’. For more information please visit please visit www.springernature.com/orcid.

We look forward to seeing the revised manuscript and thank you for the opportunity to review your work.

Sincerely,
Wei

Wei Li, PhD
Senior Editor
Nature Genetics
New York, NY 10004, USA
www.nature.com/ng

Reviewers' Comments:

Reviewer #1:
Remarks to the Author:
In this manuscript, Lian et al. report their work on Arabidopsis thaliana pan-genomics study by completing chromosome-scale genome assemblies of 72 diverse A. thaliana accessions covering a wide range of geographic distributions. The authors performed an extensive analysis of structure variation and highlight interesting results from centromeric regions using reference-quality genome assemblies compared to previous Col-0 assemblies. They also conducted pan-genome analyses, which revealed a higher number of new private genes compared to previous A. thaliana pan-genomics work. Some of their analysis and results are slightly redundant from previous work, but adding many more genomes, which is of course valuable. The paper is very well written. The data generated represent a major step forward and will undoubtedly be extremely useful to many researchers.

"Major" comments:

The authors have generated some very valuable data, but it would be more impactful and useful if some additional files were made available (e.g. via Figshare or similar). For example, it would be great if they provide the pan-genome matrix, gene family results from orthofinder, VCF for SVs, and genome annotations (in addition to the assemblies which they say will be submitted to NCBI after publication). I believe making these available will significantly improve the value of the outstanding resources generated here.

One of the major challenges in pan-genomics in general seems to arise from annotation, both of genes themselves and their functions. Can the authors help explain, for example, what is going on with Shahdara in Fig4B? There are regions that appear syntenic with other accessions but while those sequences other genomes are annotated as "clustered NLRs" the same sequence in Shahdara is annotated as "protein coding". Were these missed by NLR Annotator and RGAugury? There are other similar cases throughout the figure that suggest something funky is going on, but perhaps I am just not looking at it correctly.

I am trying to wrap my head around all the private genes found. Because the authors used sequences to define orthogroups, I am wondering how many cases of "private genes" are actually just allelic variants of a "gene" that is shared by other accessions but with this one allele that is so diverged it is clustering in its own orthogroup. For example, if an accession has an allele distinguished from the rest of the species by a portion deleted or a huge number of amino acid changes, would it show up as a private gene, even if that same "gene" is found in other accessions? Or if a gene with multiple exons is erroneously annotated as multiple genes in one accession would those new "genes" (which are in

reality just fragments of a single gene) be called as private? It would be nice if the authors could address, in the text and/or with some analyses, why such scenarios may or may not be affecting things. It might help to highlight, in Fig 4B (and perhaps generate some additional, similarly-formatted figures) which genes are annotated as private to better understand what these private genes really are.

Given the challenges of annotation, it might be good to discuss somewhere what level of skepticism/confidence we, as readers, should have about the validity of private genes. Especially since from figure 5 it seems that they are often small fragments, with half being smaller than 64 amino acids in length (5C), having 0 domains (5D), and at least half being unsupported by RNAseq (5E). How confident are the authors that some private genes are not arising from annotation issues (either in the annotation of assemblies or properly distinguishing allelic variants from truly unique "genes")? If the authors suspect that some of such challenges at least partially explain the large number of private genes, it would be good to discuss openly. This is a truly pioneering paper, with a massive increase in the number of genomes analyzed, so I think the field would benefit from a candid view of the technical challenges that had to be overcome. Rather than detracting, I think it would really make the paper more valuable so that others can think about the technical hurdles in the age of pan-genomics.

If we should be confident in the private genes, I'd be interested to know the authors' thoughts on how they arise. Are private genes duplications followed by rapid divergence (enough for them to be called as a unique family), de novo gene birth, or are these private genes the last holdouts from loss events that occurred in the rest of the species genomes? If these hypotheses (or others about the nature of the private genes) were addressed, it may elevate the impact of the paper by providing some new fundamental insights while also potentially helping alleviate concerns about the technical challenges of defining "private" genes.

One of the most interesting results from the paper is the breakdown of synteny in centromeres. I suspect this is largely biological (i.e. real) but there could be concerns that some of it is due to the difficulty of assembling in centromeres. We have found that visualizing contig breakpoints can help with identifying unreliable SVs in pangenome analyses. It would be nice if the authors added:

1)Points along the chromosomes in figure 3 (and corresponding supplemental figures for individual chromomosomes) showing the contig break points.

2)A density distribution above 4A showing the location of contig breakpoints in the assemblies.

Minor comments:

Line 192: full name of NORs?
Figure 2A: pictures overlap a bit ('155' on x-axis is cut-off)

The genomics community would probably find some value if the authors did more comparisons of genome qualities (e.g. contig N50) versus structural variants and annotation (e.g. number of private genes), perhaps coloring points by HiFi and Nanopore genomes. For example, do genomes with lower contig N50's have higher SV and more private genes?

Figure 5G: It seems that most of the pangenome is driven by private genes. How does this figure look

without those private genes?

Bit nit-picky but in the legend of FigS15, arent the colors according to "classification from Structure" rather than "phylogenetic classification"?

The authors used an interesting strategy to assemble the genome, combining results from multiple assemblers with quickmerge. It might be useful for the community (e.g. those considering citing this approach) if the authors provided some information about how well this performed. For example, which of the three assemblers gave the highest N50 (which the authors used as the starting point for the merging process), and how often did the other assemblies help in merging those contigs?

Pg11, Line 290: "30-40" should be removed.
Pg11, Line 313: "assemblys" should be corrected to "assemblies".

Overall, its a great paper, and I am excited to see it published.

Reviewer #2:
Remarks to the Author:
This is a nicely presented manuscript describing a pangenomics study that to a large degree is conservatively presented (by which I mean no slight, simply that it focuses on gene space presence/absence analysis - as have many of the pre-long read pangenomics papers, especially in bacteria - and it favors a non-graph-based approach). Accordingly, a major focus is on core, softcore, and private gene space, which is still interesting. However, I see the major novel finding as the centromere variation finding, which is timely.

While this is so, I am less sure this is a report of especially broad interest nor especially of novel or very far-reaching impact as presented. In particular, the authors would do well to make much clearer how this work goes so far beyond the studies of Kang et al 2022 and that of Wlodzimierz et al 2023 that it should be published in this very top journal. Aside from extending the centromere size variation reported in Wlodzimierz to that of overall genome size, and of generally confirming and refining Kang's pangenome gene space analysis, I see the impacts as broader sampling and better demonstrating overall genome size variation in Arabidopsis. I value these, and in particular the genome size analysis, but I am not convinced that these findings are of the broadest interest and impact in the field of genetics across systems and subdisciplines. The core observation of the manuscript of a conserved structural genomic architecture is not especially surprising or interesting, given the subject is a particularly low diversity, very highly selfing plant. Again, I do not mean these comments as a slight; it is simply not at all surprising that relative to much higher diversity species (in various kingdoms) Arabidopsis is not very diverse also at the structural genomic level. Perhaps the authors could effectively repatch this to capture the novelty and impact better. In doing so I would suggest some toning down of the hyperbolic language: one risks alienating the reader using 'remarkably' many times in a short paper: I certainly didn't think the overall findings merited four instances of this word.

Regarding methodology, the work appears sound and the presentation of the figures is particularly excellent.

Minor points:

Leading with so much short-read data is underwhelming. Is this necessary? If so, should this not immediately be better signposted and put in the context of the enormous WG-based demographic literature in this species?

Line 28: 'remarkably' as at 170 seems a bit much here also. And besides, relative to what?

36: 'vast': not really by genomic diversity terms (think vs outcrossers and non-minuscule genomes). This really should be toned down!

109: 'metrics'

166: the statement 'lines from Sweden and Col' is unclear; please rephrase.

169: 'long ago in evolutionary time' is a bit of a redundant phrase: strike the last three words?

170: 'Remarkably' seems a bit much to describe what amounts to the occasional large-scale chromosome changes we see in plant genomes.

Fig S3: please show the busco percentages and not raw numbers.

266: 'large untapped genetic diversity' compared to what?

272: 'remarkably' again. I am not convinced this is remarkable, given A.thaliana shows very low diversity relative to most plant species studied.

I'm sorry to say, but the last line pretty much sums up how this reader is left considering the findings of this manuscript. It will be a helpful dataset for future functional investigation.

Reviewer #3:
Remarks to the Author:
The authors conducted the chromosome-level assembly of 72 accessions of the model plant species Arabidopsis thaliana. A recent focus of genomics is the pan-genome analysis using multiple high-quality assemblies, reported using several species. Using Arabidopsis thaliana, there is only a preprint reporting 38 genomes, which is cited in the manuscript. Considering the importance of the model species, the report is timely. The authors found that chromosomal rearrangements in chromosome arms are rare, suggesting that the karyotype is mostly fixed in the species. The pan-genome analysis identified 40,382 distinct genes, which is drastically more than 27,445 genes in the single reference (Araport11).
Considering significance, the genome data will be an excellent resource for future studies. Pan-genome can potentially become a standard reference instead the of currently used TAIR10/Araport11 reference. The data and methodology are solid. I did not find any major issues.
I should mention that there is no surprise or no major biological finding beyond the pan-genome identification in this manuscript. In other words, the manuscript is mostly descriptive. This may be an inherent nature of genome assembly paper. I would suggest this should be considered by the editors.
As a potential improvement, some sort of quality check of the assembly would strengthen the

existence of rare rearrangements. The same rearrangement observed in more than two accessions is strongly supported. However, the authors reported that most of the large rearrangements were rare, i.e., found in a single accession. It is not feasible to distinguish between a real rare rearrangement and a rare assembly error. Do the authors expect all (or nearly all) of them to be authentic? The authors may visualize long reads across the breakpoints to examine some of the rearrangements. It would be best to show experimental evidence (for example, PCR beyond breakpoints) although it may not be feasible depending on the repetitive sequences.
The terminology of "gene families" and "genes" should be clearly defined. The abstract (line 35) said "Pan-genome analyses of the gene space uncovered 40,382 distinct genes".
Line 222 said "we found that 40,382 gene families included genes from at least one of the 72 A. thaliana genomes."
I assume that the authors referred to the same thing. Then, "gene families" in the latter sentence may not be really a gene family, but a clade of sequences composed of alleles of different accessions. Please check the manuscript thoroughly.

Minor comments
Line 48. If the authors cite the paper on 20 barley genomes, the paper on bread wheat 10 genomes (Walkowiak et al. Nature 588:277, 2020) was published on the same issue and would be better cited together. The quality of the assemblies of these large-genome species was clearly lower than those of Arabidopsis thaliana in this manuscript, but they provided significant prospects for pan-genome analysis.

Line 217 T2T would be better spelled out for the moment.

Author Rebuttal to Initial comments

Reviewer #1:

Remarks to the Author:

In this manuscript, Lian et al. report their work on Arabidopsis thaliana pan-genomics study by completing chromosome-scale genome assemblies of 72 diverse A. thaliana accessions covering a wide range of geographic distributions. The authors performed an extensive analysis of structure variation and highlight interesting results from centromeric regions using reference-quality genome assemblies compared to previous Col-0 assemblies. They also conducted pan-genome analyses, which revealed a higher number of new private genes compared to previous A. thaliana pan-genomics work. Some of their analysis and results are slightly redundant from previous work, but adding many more genomes, which is of course valuable. The paper is very well written. The data generated represent a major step forward and will undoubtedly be extremely useful to many researchers.

Thank you very much for your supportive evaluation of our work.

"Major" comments:
The authors have generated some very valuable data, but it would be more impactful and useful if some additional files were made available (e.g. via Figshare or similar). For example, it would be great if they provide the pan-genome matrix, gene family results from orthofinder, VCF for SVs, and genome

annotations (in addition to the assemblies which they say will be submitted to NCBI after publication). I believe making these available will significantly improve the value of the outstanding resources generated here.

Besides submitting the sequencing raw data to EMBL-ENA (PRJEB62038), genome assemblies to NCBI (PRJNA1033522), we now also uploaded the related files, including assemblies, gene and TE annotations, pan-genome matrix, orthogroups, and VCF files with SNPs and SVs etc to Edmond (the Open Research Data Repository of the Max Planck Society, https://edmond.mpdl.mpg.de/privateurl.xhtml?token=adea01db-8129-4c2c-942f-b99237260f19).

One of the major challenges in pan-genomics in general seems to arise from annotation, both of genes themselves and their functions. Can the authors help explain, for example, what is going on with Shahdara in Fig4B? There are regions that appear syntenic with other accessions but while those sequences other genomes are annotated as “clustered NLRs” the same sequence in Shahdara is annotated as "protein coding". Were these missed by NLR Annotator and RGAugury? There are other similar cases throughout the figure that suggest something funky is going on, but perhaps I am just not looking at it correctly.

Indeed, thank you for pointing out this mistake. The Shahdara NLRs were wrongly colored when creating this figure – in fact, these genes were also annotated as NLRs in Shahdara. This is now corrected in the revised Figure 4B.
In general, however, as described in the original papers of NLR Annotator and RGAugury, both tools cannot find all NLR genes in Arabidopsis. As such, we cannot avoid the possibility that some NLRs were missed in our analysis. Furthermore, divergent genes (with non-intact RGA-related protein domains and motifs) might also be missed in the NLR annotations.

I am trying to wrap my head around all the private genes found. Because the authors used sequences to define orthogroups, I am wondering how many cases of “private genes” are actually just allelic variants of a “gene” that is shared by other accessions but with this one allele that is so diverged it is clustering in its own orthogroup. For example, if an accession has an allele distinguished from the rest of the species by a portion deleted or a huge number of amino acid changes, would it show up as a private gene, even if that same “gene” is found in other accessions? Or if a gene with multiple exons is erroneously annotated as multiple genes in one accession would those new “genes” (which are in reality just fragments of a single gene) be called as private? It would be nice if the authors could address, in the text and/or with some analyses, why such scenarios may or may not be affecting things. It might help to highlight, in Fig 4B (and perhaps generate some additional, similarly-formatted figures) which genes are annotated as private to better understand what these private genes really are.

Given the challenges of annotation, it might be good to discuss somewhere what level of skepticism/confidence we, as readers, should have about the validity of private genes. Especially since from figure 5 it seems that they are often small fragments, with half being smaller than 64 amino acids in length (5C), having 0 domains (5D), and at least half being unsupported by RNAseq (5E). How confident are the authors that some private genes are not arising from annotation issues (either in the annotation of assemblies or properly distinguishing allelic variants from truly unique "genes")? If the authors suspect that some of such challenges at least partially explain the large number of private genes, it would be good to discuss openly. This is a truly pioneering paper, with a massive increase in the number of genomes analyzed, so I think the field would benefit from a candid view of the technical

challenges that had to be overcome. Rather than detracting, I think it would really make the paper more valuable so that others can think about the technical hurdles in the age of pan-genomics.

If we should be confident in the private genes, I'd be interested to know the authors' thoughts on how they arise. Are private genes duplications followed by rapid divergence (enough for them to be called as a unique family), de novo gene birth, or are these private genes the last holdouts from loss events that occurred in the rest of the species genomes? If these hypotheses (or others about the nature of the private genes) were addressed, it may elevate the impact of the paper by providing some new fundamental insights while also potentially helping alleviate concerns about the technical challenges of defining "private" genes.

Thank you for bringing up these important points.

Broadly speaking, orthogroups are generated by clustering genes based on their pairwise sequence similarity, and further classification (core, softcore, dispensable and private) is based on these orthogroups. Like all classifications and clustering algorithms, there are distinct decisions to cluster or to not cluster individual genes into the same orthogroups. This process can lead to the false separation of diverse alleles of the same gene. As we were also surprised by the high number of private genes, we have re-checked carefully our entire analysis, paying peculiar attention to any potential source of artefactual private genes. These analyses allowed us to eliminate some artifacts, but the number of private genes remained very large:

First, the genome assemblies of three accessions were of lower quality than the other accessions. During the revision time, we sequenced them again with a fresh DNA preparation, without solving the problem. In contrast to the expectation for a pure line, these accessions showed signs of heterozygosity (Supplemental Figure 28), suggesting segregation of heterozygous regions or contamination by another accession. The other 69 accessions were confirmed to be pure lines. We thus decided to exclude these three accessions for all the downstream analysis (but we made the raw data and the assemblies available to download).

Second, we identified some contigs that were not scaffolded into either of the five pseudo-chromosomes and whose genes showed similarity to bacterial genes. We concluded that these contigs resulted from bacterial DNA, and not from Arabidopsis. We have thus removed the corresponding genes (a total of 3646 genes, including 1388 private genes).

Third, we checked how reliable the clustering of the genes was. Especially for the private genes, we performed all-against-all comparisons against the genes of the other accessions. The private gene loci were checked for homology to other genes of other accessions. We found multiple cases, where private genes showed high similarity to genes of orthogroups in which the majority of the accessions were represented, however, no gene of the accessions with the private gene was featured in such an orthogroup. In these cases, we merged the private genes into the orthogroups. One example of this, AT2G31190 (annotated in the genome of Etna2) was originally defined as a private gene. However, in our analysis it showed high similarity with genes of the orthogroup OG0020678 (containing one gene of each of the 68 other accessions, but no gene of Etna2). We therefore merged this Etna2 gene into the orthogroup. Overall, we corrected a total of ~1,000 putatively mis-clustered orthogroups.

Finally, we also evaluated the clustering of gene models which were annotated as two (or more) genes in some genomes, while the homologous regions were annotated with one gene in other genomes (so-called split-merge cases). For example, there were two adjacent genes in Sus-1, AT5G49410 (2834 bp)

and AT5G49420 (353 bp), that both can be aligned to AT5G49710 (3212 bp) in Kar-1. However, due to an SNP that introduces an early stop codon (TTA -> TAA) in Sus-1, the gene annotation of Sus-1 includes two distinct genes. This is similar to the two private genes in Tanz-1 in Figure 4B, which have "merged" homologous gene models in other accessions. In total, these split-merge cases represented ~80% of the private genes. While it is possible that some of these cases are the result of annotation errors, split-merge cases may also reflect genuine differences in the transcriptomes with functional consequences. We thus kept them in our analysis but labeled them separately to distinguish them in Figure 5B.

For all the remaining genes in the 1281 private gene families, we couldn't find any potential homologs. These genes could be genuine, *de novo* evolved genes. For example, for the two private genes in Are-10 and Est-1 shown in Figure 4B, no sequence similarity can be detected in any of the other accessions. Overall, there are four private genes in Figure 4B, which are now marked by black triangles (as per your request).

One of the most interesting results from the paper is the breakdown of synteny in centromeres. I suspect this is largely biological (i.e. real) but there could be concerns that some of it is due to the difficulty of assembling in centromeres. We have found that visualizing contig breakpoints can help with identifying unreliable SVs in pangenome analyses. It would be nice if the authors added:

1) Points along the chromosomes in figure 3 (and corresponding supplemental figures for individual chromomosomes) showing the contig break points.
2) A density distribution above 4A showing the location of contig breakpoints in the assemblies.

Thank you for this nice suggestion to outline the quality of the assemblies. We have now updated Figure 3, Supplementary Figure 7-11 (breakpoints indicated by black triangles), and Figure 4A (the chromosomal distribution indicated by the purple line). Indeed, we found an increased frequency of contig breakpoints in the centromeres, which, however, is not due to a general increase of contig breaks in all assemblies, but is dominated by the increased contig breaks in some of the assemblies.
The repetitive nature of centromeres also introduces a big challenge for the whole-genome alignment and synteny detection further complicating the analysis of these regions, even if well assembled. However, Wlodzimierz et al 2023 assembled the centromere of 66 accessions and also revealed a remarkable diversity.

Minor comments:

Line 192: full name of NORs?

Added.

Figure 2A: pictures overlap a bit ('155' on x-axis is cut-off)

Done.

The genomics community would probably find some value if the authors did more comparisons of genome qualities (e.g. contig N50) versus structural variants and annotation (e.g. number of private

genes), perhaps coloring points by HiFi and Nanopore genomes. For example, do genomes with lower contig N50's have higher SV and more private genes?

We performed a correlation analysis between contig N50 values and the number of SVs and private genes. Both analyses showed weak negative correlations (Pearson's correlation, R2 of -0.27 and -0.3) implying that assemblies with lower contig N50 tend to have more SVs and private genes.

Figure 5G: It seems that most of the pangenome is driven by private genes. How does this figure look without those private genes?

This is true, we added Supplementary Figure 26 showing a pangenome analysis without private genes, where the pan-genome size approached a plateau.

Bit nit-picky but in the legend of FigS15, arent the colors according to "classification from Structure" rather than "phylogenetic classification"?

Yes, this is corrected in the revised manuscript.

The authors used an interesting strategy to assemble the genome, combining results from multiple assemblers with quickmerge. It might be useful for the community (e.g. those considering citing this approach) if the authors provided some information about how well this performed. For example, which of the three assemblers gave the highest N50 (which the authors used as the starting point for the merging process), and how often did the other assemblies help in merging those contigs?

Yes, we added Supplementary Tables 2-3, which include the genome statistics for the initial assembly and the assembly after merge, and the selected start assembly and merge strategy. Typically, Hifiasm gives the initial assembly with the highest N50, which was selected as the start assembly. However, in 7 accessions, it generated a lower assembly size or N50 or misassembly, etc, thus the assembly from Canu or Flye was chose as the start assembly. Compared with the selected initial assembly, an increase of the N50 value can be seen for 23 of 45 and 23 of 24 accessions sequenced by PacBio HiFi and Oxford Nanopore respectively. We also updated the corresponding method part to make it clearer.

Pg11, Line 290: "30-40" should be removed.

This indicates the number of plants used to produce the DNA. This is important information and we edited the sentence to make it clearer.

Pg11, Line 313: "assemblys" should be corrected to "assemblies".

Corrected.

Overall, its a great paper, and I am excited to see it published.

Thank you again for your support.

Reviewer #2:

Remarks to the Author:

This is a nicely presented manuscript describing a pangenomics study that to a large degree is conservatively presented (by which I mean no slight, simply that it focuses on gene space presence/absence analysis - as have many of the pre-long read pangenomics papers, especially in bacteria - and it favors a non-graph-based approach). Accordingly, a major focus is on core, softcore, and private gene space, which is still interesting. However, I see the major novel finding as the centromere variation finding, which is timely.

While this is so, I am less sure this is a report of especially broad interest nor especially of novel or very far-reaching impact as presented. In particular, the authors would do well to make much clearer how this work goes so far beyond the studies of Kang et al 2022 and that of Wlodzimierz et al 2023 that it should be published in this very top journal. Aside from extending the centromere size variation reported in Wlodzimierz to that of overall genome size, and of generally confirming and refining Kang's pangenome gene space analysis, I see the impacts as broader sampling and better demonstrating overall genome size variation in Arabidopsis. I value these, and in particular the genome size analysis,

but I am not convinced that these findings are of the broadest interest and impact in the field of genetics across systems and subdisciplines. The core observation of the manuscript of a conserved structural genomic architecture is not especially surprising or interesting, given the subject is a particularly low diversity, very highly selfing plant. Again, I do not mean these comments as a slight; it is simply not at all surprising that relative to much higher diversity species (in various kingdoms) Arabidopsis is not very diverse also at the structural genomic level. Perhaps the authors could effectively repatch this to capture the novelty and impact better. In doing so I would suggest some toning down of the hyperbolic language: one risks alienating the reader using 'remarkably' many times in a short paper: I certainly didn't think the overall findings merited four instances of this word.

Regarding methodology, the work appears sound and the presentation of the figures is particularly excellent.

Thank you very much for evaluating our work and we are pleased to read about your interest in our approach and results.

In comparison to the two related manuscripts, one key contribution of our study is the analysis of a much larger and more diverse biological sample set, covering more of the geographical and genetic distribution of the species. Moreover, the study of Wlodzimierz et al 2023 is very different, mainly focusing on centromere dynamics. The study of Kang et al (published very recently, on October 06 2023) is more similar to our study, but still with some key differences. Most notably, our advanced analysis of synteny shown in Figure 3 has no equivalent in Kang et al. This analysis shows a very conserved genome structure, and we respectfully disagree that it was a given that individuals from Tanzania, the Canary Islands, and Japan would show such a similar genome organization. We also show that the variation in genome size is mostly driven by centromere repeats variation and only marginally by TEs. The pangenome analyses of Kang et al and ours are comparable, and the added value of our study is provided by a larger and more diverse sampling (more than twice in total, and more than 3 times for the diverse African clade).
We agree with the reviewer to tone down the related description in the manuscript, and notably to remove

the multiple instances of “remarkably”. We note here that our over-usage of this word might be due to a different connotation of the equivalent of “remarkably” in other languages (e.g. *remarquablement* in French), which means more “of particular interest” rather than “extraordinarily”.

Minor points:

Leading with so much short-read data is underwhelming. Is this necessary? If so, should this not immediately be better signposted and put in the context of the enormous WG-based demographic literature in this species?

We believe this is necessary, as we use this analysis to describe the genetic diversity of our sample set. However, we reduced the size of the paragraph in the revised manuscript.

Line 28: 'remarkably' as at 170 seems a bit much here also. And besides, relative to what?

Corrected

36: 'vast': not really by genomic diversity terms (think vs outcrossers and non-minuscule genomes). This really should be toned down!

Agreed. Deleted.

109: 'metrics'

Done.

166: the statement 'lines from Sweden and Col' is unclear; please rephrase.

Corrected.

169: 'long ago in evolutionary time' is a bit of a redundant phrase: strike the last three words?
Done.

170: 'Remarkably' seems a bit much to describe what amounts to the occasional large-scale chromosome changes we see in plant genomes.

Corrected.

Fig S3: please show the busco percentages and not raw numbers.

Done.

266: 'large untapped genetic diversity' compared to what?

Deleted.

272: 'remarkably' again. I am not convinced this is remarkable, given A.thaliana shows very low diversity relative to most plant species studied.

Edited.

I'm sorry to say, but the last line pretty much sums up how this reader is left considering the findings of this manuscript. It will be a helpful dataset for future functional investigation.

As discussed above, we believe that the presented results are important, but in parallel, we do not disregard the contribution of this work as a base for future research.

Reviewer #3:

Remarks to the Author:

The authors conducted the chromosome-level assembly of 72 accessions of the model plant species Arabidopsis thaliana. A recent focus of genomics is the pan-genome analysis using multiple high-quality assemblies, reported using several species. Using Arabidopsis thaliana, there is only a preprint reporting 38 genomes, which is cited in the manuscript. Considering the importance of the model species, the report is timely. The authors found that chromosomal rearrangements in chromosome arms are rare, suggesting that the karyotype is mostly fixed in the species. The pan-genome analysis identified 40,382 distinct genes, which is drastically more than 27,445 genes in the single reference (Araport11).
Considering significance, the genome data will be an excellent resource for future studies. Pan-genome can potentially become a standard reference instead the of currently used TAIR10/Araport11 reference. The data and methodology are solid. I did not find any major issues.

I should mention that there is no surprise or no major biological finding beyond the pan-genome identification in this manuscript. In other words, the manuscript is mostly descriptive. This may be an inherent nature of genome assembly paper. I would suggest this should be considered by the editors.

Thank you very much for taking the time to evaluate the work. We are pleased that you point out the quality of the data and the analyses, and that the data will be useful for many researchers.

As a potential improvement, some sort of quality check of the assembly would strengthen the existence of rare rearrangements. The same rearrangement observed in more than two accessions is strongly supported. However, the authors reported that most of the large rearrangements were rare, i.e., found in a single accession. It is not feasible to distinguish between a real rare rearrangement and a rare assembly error. Do the authors expect all (or nearly all) of them to be authentic? The authors may visualize long reads across the breakpoints to examine some of the rearrangements. It would be best to show experimental evidence (for example, PCR beyond breakpoints) although it may not be feasible depending on the repetitive sequences.

Thank you, this is a very good suggestion. To validate all six megabase-scale inversions in Shahdara, Zal-1, N13, Ws-4, Stw-0, and Ge-0, we provide Supplementary Figure 13-18 with visualizations of long-reads alignments that span over both breakpoints. In addition to the alignments against the genome where the inversion was identified, we also show the alignments of the same reads against the orthologous regions in the Col-0 genome and the closest-related accession (where the inversions are not present). All six inversions are well supported, as split read alignments can be observed in

alignments against Col and the closest accession, but not in the alignments against the genomes where the inversions were annotated.

The terminology of "gene families" and "genes" should be clearly defined. The abstract (line 35) said "Pan-genome analyses of the gene space uncovered 40,382 distinct genes". Line 222 said "we found that 40,382 gene families included genes from at least one of the 72 A. thaliana genomes." I assume that the authors referred to the same thing. Then, "gene families" in the latter sentence may not be really a gene family, but a clade of sequences composed of alleles of different accessions. Please check the manuscript thoroughly.

Thank you for pointing this out. Yes, indeed, they are two distinct terms. A “gene family” was defined by the clustering algorithm, as a group/cluster of “genes” with similarity. We made this clearer in the revised manuscript.

Minor comments
Line 48. If the authors cite the paper on 20 barley genomes, the paper on bread wheat 10 genomes (Walkowiak et al. Nature 588:277, 2020) was published on the same issue and would be better cited together. The quality of the assemblies of these large-genome species was clearly lower than those of Arabidopsis thaliana in this manuscript, but they provided significant prospects for pan-genome analysis.

Yes, this is now included.

Line 217 T2T would be better spelled out for the moment.

Corrected.

Decision Letter, first revision:

Our ref: NG-A62638R

5th Jan 2024

Dear Dr. Mercier,

Thank you for submitting your revised manuscript "A pan-genome of 69 Arabidopsis thaliana accessions reveals a conserved genome structure throughout the global species range" (NG-A62638R). It has now been seen by the original referees and their comments are below. The reviewers find that the paper has improved in revision, and therefore we'll be happy in principle to publish it in Nature Genetics, pending minor revisions to satisfy the referees' final requests and to comply with our editorial and formatting guidelines.

We are now performing detailed checks on your paper and will send you a checklist detailing our editorial and formatting requirements soon. Please do not upload the final materials and make any revisions until you receive this additional information from us.

Thank you again for your interest in Nature Genetics Please do not hesitate to contact me if you have any questions.

Sincerely,
Wei

Wei Li, PhD
Senior Editor
Nature Genetics
New York, NY 10004, USA
www.nature.com/ng

Reviewer #1 (Remarks to the Author):

The authors have done a thorough job responding to my previous comments/concerns.

They adequately addressed my concerns regarding the nature of private genes, and I found their analyses quite innovative. Their approaches to collapsing orthogroups and distinguishing private genes derived from split gene annotations provide a valuable roadmap for future pan-genome studies across organisms and yield novel insights into the challenges of defining a "pan-genome."

I also appreciate the additional datasets the authors made public. These will certainly elevate the impact of the paper and value for the broader community.

Lastly, I appreciate the author's consideration of adding contig breakpoints to the synteny metaplot, which helps provide some additional information to readers. The authors' conclusions about low synteny around centromeres being largely biological seem sound, and their updated figure helps make this clearer with appropriate context.

I am sympathetic to comments raised by other reviewers that the paper provides limited significant novel biological discoveries but remain enthusiastic about this paper given 1) the value of the resources generated to empower future research and 2) the novel approaches (such as the newly refined analyses of orthogroups and split genes) which will be informative for future pan-genome efforts broadly. Thus, I am excited to see this published and expect the larger research community will appreciate it.

Reviewer #2 (Remarks to the Author):

Overall I have only the most minor suggestions. A very nice paper!

Also: reading the responses and actions taken in response to R1 was a pleasure. The authors were responsive and the paper improved nicely.

Ln31: remove comma

Ln74: period missing after ‘analysis’ in my copy
Ln112-114: interesting finding: is there any way to indicate the ONT assemblies in Fig2A and/or SFig5?
Ln 139: centromeres to singular
157: reads to singular
Are Fig 4C and S21 identical? Except for one dark blue box in the top left of S21 and some minor overall shading difference? It would be nice to add some detail on how they differ somewhere.
277: ‘Unprecedented resource’ is quite a tall statement in the day of pangenomes much larger than this one. Rather than bristle the reader, perhaps ‘very powerful resource’ or the like could be used?

Reviewer #3 (Remarks to the Author):

The authors provided a significant amount of additional analyses in response to three reviewers' comments. They addressed all of my concerns, as detailed below (shown with >>). I have no more comments.

Reviewer #3:
Remarks to the Author:
The authors conducted the chromosome-level assembly of 72 accessions of the model plant species Arabidopsis thaliana. A recent focus of genomics is the pan-genome analysis using multiple high-quality assemblies, reported using several species. Using Arabidopsis thaliana, there is only a preprint reporting 38 genomes, which is cited in the manuscript. Considering the importance of the model species, the report is timely. The authors found that chromosomal rearrangements in chromosome arms are rare, suggesting that the karyotype is mostly fixed in the species. The pan-genome analysis identified 40,382 distinct genes, which is drastically more than 27,445 genes in the single reference (Araport11).
Considering significance, the genome data will be an excellent resource for future studies. Pan-genome can potentially become a standard reference instead the of currently used TAIR10/Araport11 reference. The data and methodology are solid. I did not find any major issues.
I should mention that there is no surprise or no major biological finding beyond the pan-genome identification in this manuscript. In other words, the manuscript is mostly descriptive. This may be an inherent nature of genome assembly paper. I would suggest this should be considered by the editors.

>Thank you very much for taking the time to evaluate the work. We are pleased that you point out the quality of the data and the analyses, and that the data will be useful for many researchers.

>>Fine from the viewpoint of a reviewer.

As a potential improvement, some sort of quality check of the assembly would strengthen the existence of rare rearrangements. The same rearrangement observed in more than two accessions is strongly supported. However, the authors reported that most of the large rearrangements were rare, i.e., found in a single accession. It is not feasible to distinguish between a real rare rearrangement and a rare assembly error. Do the authors expect all (or nearly all) of them to be authentic? The authors may visualize long reads across the breakpoints to examine some of the rearrangements. It

would be best
to show experimental evidence (for example, PCR beyond breakpoints) although it may not be feasible depending on the repetitive sequences.

>Thank you, this is a very good suggestion. To validate all six megabase-scale inversions in Shahdara, Zal-1, N13, Ws-4, Stw-0, and Ge-0, we provide Supplementary Figure 13-18 with visualizations of long- reads alignments that span over both breakpoints. In addition to the alignments against the genome where the inversion was identified, we also show the alignments of the same reads against the orthologous regions in the Col-0 genome and the closest-related accession (where the inversions are not present). All six inversions are well supported, as split read alignments can be observed in alignments against Col and the closest accession, but not in the alignments against the genomes where the inversions were annotated.

>>A similar point was also raised by the Reviewer 1. New figures are satisfactory.

The terminology of "gene families" and "genes" should be clearly defined. The abstract (line 35) said "Pan-genome analyses of the gene space uncovered 40,382 distinct genes". Line 222 said "we found that 40,382 gene families included genes from at least one of the 72 A. thaliana genomes." I assume that the authors referred to the same thing. Then, "gene families" in the latter sentence may not be really a gene family, but a clade of sequences composed of alleles of different accessions. Please check the manuscript thoroughly.

>Thank you for pointing this out. Yes, indeed, they are two distinct terms. A "gene family" was defined by the clustering algorithm, as a group/cluster of "genes" with similarity. We made this clearer in the revised manuscript.

>>Fine.

Minor comments
Line 48. If the authors cite the paper on 20 barley genomes, the paper on bread wheat 10 genomes (Walkowiak et al. Nature 588:277, 2020) was published on the same issue and would be better cited together. The quality of the assemblies of these large-genome species was clearly lower than those of Arabidopsis thaliana in this manuscript, but they provided significant prospects for pan-genome analysis.

>Yes, this is now included.

>>Fine.

Line 217 T2T would be better spelled out for the moment.

>Corrected.

>>Fine.

Author Rebuttal, first revision:

Reviewer #1:
Remarks to the Author:
The authors have done a thorough job responding to my previous comments/concerns.

They adequately addressed my concerns regarding the nature of private genes, and I found their analyses quite innovative. Their approaches to collapsing orthogroups and distinguishing private genes derived from split gene annotations provide a valuable roadmap for future pan-genome studies across organisms and yield novel insights into the challenges of defining a "pan-genome."

I also appreciate the additional datasets the authors made public. These will certainly elevate the impact of the paper and value for the broader community.

Lastly, I appreciate the author's consideration of adding contig breakpoints to the synteny metaplot, which helps provide some additional information to readers. The authors' conclusions about low synteny around centromeres being largely biological seem sound, and their updated figure helps make this clearer with appropriate context.

I am sympathetic to comments raised by other reviewers that the paper provides limited significant novel biological discoveries but remain enthusiastic about this paper given 1) the value of the resources generated to empower future research and 2) the novel approaches (such as the newly refined analyses of orthogroups and split genes) which will be informative for future pan-genome efforts broadly. Thus, I am excited to see this published and expect the larger research community will appreciate it.

We are pleased that the revision improved the manuscript and thank the reviewers for the detailed review and the suggestions for further improvement of clarity. Thank you very much for pointing out the high relevance of our work.

Reviewer #2:
Remarks to the Author:
Overall I have only the most minor suggestions. A very nice paper! Thank you!

Also: reading the responses and actions taken in response to R1 was a pleasure. The authors were responsive and the paper improved nicely.

Ln31: remove comma
Ln74: period missing after 'analysis' in my copy We have

corrected the text accordingly.

Ln112-114: interesting finding: is there any way to indicate the ONT assemblies in Fig2A and/or SFig5?

Thanks for your suggestion. We have added the information of sequencing technologies in Supplemantaty Figure 5 (now Extended Data Figure 2). Besides, all related source data can be found in Source Data Figure 2 and Supplementaty Tables.

Ln 139: centromeres to singular 157:

reads to singular

We have corrected the text accordingly.

Are Fig 4C and S21 identical? Except for one dark blue box in the top left of S21 and some minor overall shading difference? It would be nice to add some detail on how they differ somewhere.

Figure 4c is the comparison across chromosome arms, while Figure S21 is across the whole chromosome. We have rephrased the Figure title and legend to make it more clear.

277: ‘Unprecedented resource’ is quite a tall statement in the day of pangenomes much larger than this one. Rather than bristle the reader, perhaps ‘very powerful resource’ or the like could be used?

We have corrected the text accordingly.

Reviewer #3:
Remarks to the Author:
The authors provided a significant amount of additional analyses in response to three reviewers' comments. They addressed all of my concerns, as detailed below (shown with >>). I have no more comments.

Reviewer #3:
Remarks to the Author:
The authors conducted the chromosome-level assembly of 72 accessions of the model plant species Arabidopsis thaliana. A recent focus of genomics is the pan-genome analysis using multiple high-quality assemblies, reported using several species. Using Arabidopsis thaliana, there is only a preprint reporting 38 genomes, which is cited in the manuscript. Considering the importance of the model species, the report is timely. The authors found that chromosomal rearrangements in chromosome arms are rare, suggesting that the karyotype is mostly fixed in the species. The pan-genome analysis identified 40,382 distinct genes, which is drastically more than 27,445 genes in the single reference (Araport11).
Considering significance, the genome data will be an excellent resource for future studies. Pan-genome can potentially become a standard reference instead the of currently used TAIR10/Araport11 reference. The data and methodology are solid. I did not find any major issues.
I should mention that there is no surprise or no major biological finding beyond the pan- genome identification in this manuscript. In other words, the manuscript is mostly descriptive. This may be an inherent nature of genome assembly paper. I would suggest this should be considered by the editors.

>Thank you very much for taking the time to evaluate the work. We are pleased that you point out the quality of the data and the analyses, and that the data will be useful for many researchers.

>>Fine from the viewpoint of a reviewer.

As a potential improvement, some sort of quality check of the assembly would strengthen the existence of rare rearrangements. The same rearrangement observed in more than two accessions is strongly supported. However, the authors reported that most of the large rearrangements were rare, i.e., found in a single accession. It is not feasible to distinguish between a real rare rearrangement and a rare assembly error. Do the authors expect all (or nearly all) of them to be authentic? The authors may

visualize long reads across the breakpoints to examine some of the rearrangements. It would be best to show experimental evidence (for example, PCR beyond breakpoints) although it may not be feasible depending on the repetitive sequences.

>Thank you, this is a very good suggestion. To validate all six megabase-scale inversions in Shahdara, Zal-1, N13, Ws-4, Stw-0, and Ge-0, we provide Supplementary Figure 13-18 with visualizations of long- reads alignments that span over both breakpoints. In addition to the alignments against the genome where the inversion was identified, we also show the alignments of the same reads against the orthologous regions in the Col-0 genome and the closest-related accession (where the inversions are not present). All six inversions are well supported, as split read alignments can be observed in alignments against Col and the closest accession, but not in the alignments against the genomes where the inversions were annotated.

>>A similar point was also raised by the Reviewer 1. New figures are satisfactory.

The terminology of "gene families" and "genes" should be clearly defined. The abstract (line 35) said "Pan-genome analyses of the gene space uncovered 40,382 distinct genes". Line 222 said "we found that 40,382 gene families included genes from at least one of the 72 A. thaliana genomes." I assume that the authors referred to the same thing. Then, "gene families" in the latter sentence may not be really a gene family, but a clade of sequences composed of alleles of different accessions. Please check the manuscript thoroughly.

>Thank you for pointing this out. Yes, indeed, they are two distinct terms. A “gene family” was defined by the clustering algorithm, as a group/cluster of “genes” with similarity. We made this clearer in the revised manuscript.

>>Fine.

Minor comments
Line 48. If the authors cite the paper on 20 barley genomes, the paper on bread wheat 10 genomes (Walkowiak et al. Nature 588:277, 2020) was published on the same issue and would be better cited together. The quality of the assemblies of these large-genome species was clearly lower than those of Arabidopsis thaliana in this manuscript, but they provided significant prospects for pan-genome analysis.

>Yes, this is now included.

>>Fine.

Line 217 T2T would be better spelled out for the moment.

>Corrected.
>>Fine.

Thank you very much for reviewing our work.

Final Decision Letter:

natureresearch

11th Mar 2024

Dear Dr. Mercier,

I am delighted to say that your manuscript "A pan-genome of 69 Arabidopsis thaliana accessions reveals a conserved genome structure throughout the global species range" has been accepted for publication in an upcoming issue of Nature Genetics.

Over the next few weeks, your paper will be copyedited to ensure that it conforms to Nature Genetics style. Once your paper is typeset, you will receive an email with a link to choose the appropriate publishing options for your paper and our Author Services team will be in touch regarding any additional information that may be required.

After the grant of rights is completed, you will receive a link to your electronic proof via email with a request to make any corrections within 48 hours. If, when you receive your proof, you cannot meet this deadline, please inform us at rjsproduction@springernature.com immediately.

You will not receive your proofs until the publishing agreement has been received through our system.

Due to the importance of these deadlines, we ask that you please let us know now whether you will be difficult to contact over the next month. If this is the case, we ask you provide us with the contact information (email, phone and fax) of someone who will be able to check the proofs on your behalf, and who will be available to address any last-minute problems.

Your paper will be published online after we receive your corrections and will appear in print in the next available issue. You can find out your date of online publication by contacting the Nature Press Office (press@nature.com) after sending your e-proof corrections.

You may wish to make your media relations office aware of your accepted publication, in case they consider it appropriate to organize some internal or external publicity. Once your paper has been scheduled you will receive an email confirming the publication details. This is normally 3-4 working days in advance of publication. If you need additional notice of the date and time of publication, please let the production team know when you receive the proof of your article to ensure there is sufficient time to coordinate. Further information on our embargo policies can be found here: https://www.nature.com/authors/policies/embargo.html

Before your paper is published online, we shall be distributing a press release to news organizations worldwide, which may very well include details of your work. We are happy for your institution or funding agency to prepare its own press release, but it must mention the embargo date and Nature Genetics. Our Press Office may contact you closer to the time of publication, but if you or your Press Office have any enquiries in the meantime, please contact press@nature.com.

Acceptance is conditional on the data in the manuscript not being published elsewhere, or announced in the print or electronic media, until the embargo/publication date. These restrictions are not intended to deter you from presenting your data at academic meetings and conferences, but any enquiries from the media about papers not yet scheduled for publication should be referred to us.

Please note that *Nature Genetics* is a Transformative Journal (TJ). Authors may publish their research

with us through the traditional subscription access route or make their paper immediately open access through payment of an article-processing charge (APC). Authors will not be required to make a final decision about access to their article until it has been accepted. Find out more about Transformative Journals

Authors may need to take specific actions to achieve compliance with funder and institutional open access mandates. If your research is supported by a funder that requires immediate open access (e.g. according to Plan S principles) then you should select the gold OA route, and we will direct you to the compliant route where possible. For authors selecting the subscription publication route, the journal's standard licensing terms will need to be accepted, including <a href="https://www.nature.com/nature-portfolio/editorial-policies/self-archiving-and-license-to-publish. Those licensing terms will supersede any other terms that the author or any third party may assert apply to any version of the manuscript.

If you have any questions about our publishing options, costs, Open Access requirements, or our legal forms, please contact ASJournals@springernature.com

If you have posted a preprint on any preprint server, please ensure that the preprint details are updated with a publication reference, including the DOI and a URL to the published version of the article on the journal website.

To assist our authors in disseminating their research to the broader community, our SharedIt initiative provides you with a unique shareable link that will allow anyone (with or without a subscription) to read the published article. Recipients of the link with a subscription will also be able to download and print the PDF.

As soon as your article is published, you will receive an automated email with your shareable link.

You can now use a single sign-on for all your accounts, view the status of all your manuscript submissions and reviews, access usage statistics for your published articles and download a record of your refereeing activity for the Nature journals.

An online order form for reprints of your paper is available at https://www.nature.com/reprints/author-reprints.html. Please let your coauthors and your institutions' public affairs office know that they are also welcome to order reprints by this method.

If you have not already done so, we invite you to upload the step-by-step protocols used in this manuscript to the Protocols Exchange, part of our on-line web resource, natureprotocols.com. If you complete the upload by the time you receive your manuscript proofs, we can insert links in your article that lead directly to the protocol details. Your protocol will be made freely available upon publication of your paper. By participating in natureprotocols.com, you are enabling researchers to more readily reproduce or adapt the methodology you use. Natureprotocols.com is fully searchable, providing your protocols and paper with increased utility and visibility. Please submit your protocol to https://protocolexchange.researchsquare.com/. After entering your nature.com username and password you will need to enter your manuscript number (NG-A62638R1). Further information can be found at https://www.nature.com/nature-portfolio/editorial-policies/reporting-standards#protocols

Sincerely,
Wei

Wei Li, PhD
Senior Editor
Nature Genetics
New York, NY 10004, USA
www.nature.com/ng
